# The palmitoyl-CoA ligase Fum16 is part of a *Fusarium verticillioides* fumonisin subcluster involved in self-protection

**DOI:** 10.1128/mbio.02681-24

**Published:** 2024-12-20

**Authors:** Fabio Gherlone, Katarina Jojić, Ying Huang, Sandra Hoefgen, Vito Valiante, Slavica Janevska

**Affiliations:** 1 (Epi-)Genetic Regulation of Fungal Virulence, Leibniz Institute for Natural Product Research and Infection Biology-Hans Knöll Institute (Leibniz-HKI), Jena, Germany; 2 Biobricks of Microbial Natural Product Syntheses, Leibniz Institute for Natural Product Research and Infection Biology-Hans Knöll Institute (Leibniz-HKI), Jena, Germany; The University of British Columbia, Vancouver, British Columbia, Canada

**Keywords:** *Fusarium*, fumonisin B_1_, *FUM *cluster, self-protection, ceramide biosynthesis, palmitoyl-CoA ligase

## Abstract

*Fusarium verticillioides* produces the mycotoxin fumonisin B_1_ (FB_1_), which disrupts sphingolipid biosynthesis by inhibiting ceramide synthase and affects the health of plants, animals, and humans. The means by which *F. verticillioides* protects itself from its own mycotoxin are not completely understood. Some fumonisin (*FUM*) cluster genes do not contribute to the biosynthesis of the compound, but their function has remained enigmatic. Recently, we showed that *FUM17*, *FUM18,* and *FUM19* encode two ceramide synthases and an ATP-binding cassette transporter, respectively, which play a role in antagonizing the toxicity mediated by FB_1_. In the present work, we uncovered functions of two adjacent genes, *FUM15* and *FUM16*. Using homologous and heterologous expression systems, in *F. verticillioides* and *Saccharomyces cerevisiae*, respectively, we provide evidence that both contribute to protection against FB_1_. Our data indicate a potential role for the P450 monooxygenase Fum15 in the modification and detoxification of FB_1_ since the deletion and overexpression of the respective gene affected extracellular FB_1_ levels in both hosts. Furthermore, relative quantification of ceramide intermediates and an *in vitro* enzyme assay revealed that Fum16 is a functional palmitoyl-CoA ligase. It co-localizes together with the ceramide synthase Fum18 to the endoplasmic reticulum, where they contribute to sphingolipid biosynthesis. Thereby, *FUM15-19* constitute a subcluster within the *FUM* biosynthetic gene cluster dedicated to the fungal self-protection against FB_1_.

**IMPORTANCE:**

The study identifies a five-gene *FUM* subcluster (*FUM15-19*) in *Fusarium verticillioides* involved in self-protection against FB_1_. *FUM16* (palmitoyl-CoA ligase), *FUM17,* and *FUM18* (ceramide synthases) enzymatically supplement ceramide biosynthesis, while *FUM19* (ATP-binding cassette transporter) acts as a repressor of the *FUM* cluster. The evolutionary conservation of *FUM15* (P450 monooxygenase) in *Fusarium* and *Aspergillus FUM* clusters is discussed, and its effect on extracellular FB_1_ levels in both native (*F. verticillioides*) and heterologous (*Saccharomyces cerevisiae*) hosts is highlighted. These findings enhance our understanding of mycotoxin self-protection mechanisms and could inform strategies for predicting biological activity of unknown secondary metabolites, managing mycotoxin contamination, and developing resistant crop cultivars.

## INTRODUCTION

Fungal secondary metabolites (SMs) are diverse organic compounds that are not essential for growth or reproduction but provide ecological advantages to the organism. These compounds help deter predators, inhibit competitors, and facilitate symbiotic relationships, enhancing the organism’s survival, reproduction, and adaptability under diverse environmental conditions (1–4).

Among the different SMs produced by fungi, mycotoxins are a significant group with a profound impact on the health of all living organisms, that is, microbes, plants, animals, and humans. They can contaminate food and feed, being produced by phytopathogenic fungi, which can lead to severe health issues in humans, like cancer, liver damage, and immunosuppression (5). Fungi exhibit remarkable resilience against self-intoxication by their own mycotoxins, an essential trait for their survival and ecological success. Mechanisms underlying this resistance include (i) efficient efflux pumps that secrete toxins from cells (6); (ii) enzymatic detoxification pathways (7); (iii) duplicated or resistant target enzymes (8); and (iv) subcellular separation of the toxic product (9). The biosynthetic gene clusters (BGCs) responsible for the production of mycotoxins often carry adjacent non-biosynthetic genes that are relevant for conferring self-protection (10).

**FIG 1:**
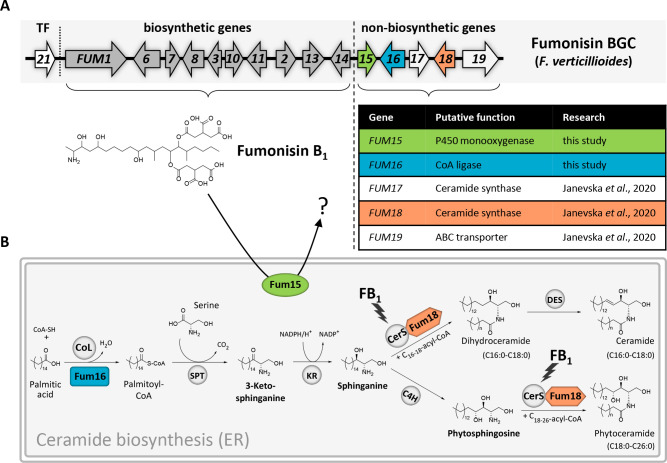
The fumonisin gene cluster and its effects on ceramide biosynthesis. (**A**) The *FUM* biosynthetic gene cluster in *F. verticillioides*. The genes encoding FB_1_ biosynthetic enzymes *FUM1–FUM14* are colored gray; the transcription factor (TF) *FUM21* is reported in white; the non-biosynthetic genes *FUM15*, *FUM16*, and *FUM18* are colored green, cyan, and orange, respectively. The table summarizes the function of *FUM* non-biosynthetic genes. (**B**) The simplified *de novo* synthesis of fungal ceramides. Indicated are catalyzing enzymes fatty acid CoA ligase (CoL), serine palmitoyltransferase (SPT), 3-ketoreductase (KR), C4-hydroxylase (C4H), desaturase (DES), and ceramide synthase (CerS). Ceramide intermediates analyzed in this study by HPLC-HRMS are written in bold. The *FUM* non-biosynthetic enzymes supplementing the pathway are included: Fum16 (cyan) and Fum18 (orange).

The sphingolipid biosynthesis inhibitors fumonisins are a group of mycotoxins produced primarily by *Fusarium* and *Aspergillus* species (11). They are polyketide-derived aminopentol compounds with a linear C_20_ backbone and two tricarballylic esters. The best studied are B-series fumonisins (FBs), FB_1_, FB_2_, FB_3_, and FB_4_, which are predominantly produced by the corn-infecting fungus *Fusarium verticillioides*. As demonstrated in mice experiments, the most toxic FB analog is FB_1_, which is the final biosynthetic product accumulating in greatest abundance in *F. verticillioides* (approximately 80%) (12–14). The fumonisin BGC (*FUM*) includes 16 genes (Fig. 1A) (12), expressed under the control of the Zn(II)_2_Cys_6_-type transcription factor Fum21 (15).

FB_1_ biosynthesis begins with the formation of the polyketide backbone by the polyketide synthase Fum1 (16) (Fig. S1). Next, the aminotransferase Fum8 catalyzes the condensation of the polyketide with l-alanine (17). Further modifications are performed by Fum6 (18), Fum13 (19), and Fum2 (20). The tricarballylic acid side chains are synthesized by Fum7 and Fum10 (21), and are attached to the backbone by Fum14 (22). FB_1_ biosynthesis ends with the hydroxylation at the C-5 position by Fum3 (23) (Fig. S1).

FB_1_ disrupts sphingolipid metabolism by inhibiting ceramide synthase (CerS), a key enzyme in ceramide biosynthesis (Fig. 1B) (24). Because FB_1_ is structurally similar to sphinganine, a substrate in ceramide biosynthesis, it competitively inhibits the enzyme by irreversibly binding to the active site of CerS (25). The inhibition of CerS by sphinganine analog mycotoxins triggers apoptosis, which is caused by an accumulation of sphingoid bases, rather than reduced sphingolipid biosynthesis itself (26). Targeted application of sphingolipid biosynthesis inhibitors, however, has promising potential as treatment against severe human diseases, such as cancer, schizophrenia, Alzheimer’s, multiple sclerosis, and diabetes (27–29).

Ceramides constitute the backbone of complex sphingolipids. In filamentous fungi, ceramide biosynthesis begins with the condensation of l-serine with palmitoyl-CoA by a serine palmitoyltransferase to form 3-ketosphinganine, which is further reduced by a ketoreductase to sphinganine. At this point, ceramide biosynthesis branches (30). Sphinganine is converted by CerS to dihydroceramide, and subsequently to ceramide by a desaturase. Alternatively, sphinganine is converted to phytosphingosine by a C4-hydroxylase and to phytoceramide by CerS (Fig. 1B). Ceramides are vital components of cell membranes and signaling molecules involved in apoptosis, cell differentiation, and proliferation (31). This disruption thus contributes to various diseases in humans and animals (32–34).

The biosynthesis of sphingolipids is compartmentalized. The first enzymatic reactions, leading up to ceramides, are catalyzed in the endoplasmic reticulum (ER), while complex sphingolipids are further synthesized in ER-derived vesicles and the Golgi (35, 36). Recently, Fum3 (the enzyme catalyzing the last reaction in FB_1_ biosynthesis) was shown to be localized in the cytosol in *F. verticillioides* (8, 37). This finding suggests a subcellular separation of the toxin FB_1_ from its target CerS in the ER, contributing to self-protection.

The *FUM* cluster in *F. verticillioides* entails five non-biosynthetic genes adjacent to one another (Fig. 1A), *FUM15* to *FUM19*. Fum19 is an ATP-binding cassette (ABC) transporter that acts as a repressor of the *FUM* gene cluster, regulating the levels of intracellular and secreted FB_1_ (8). Fum17 and Fum18 are two of five CerS homologs in *F. verticillioides*, both co-localizing with ceramide biosynthesis in the ER. In particular, Fum18 was shown to confer resistance to FB_1_ self-toxicity (8). While FB_1_ has antifungal activity, specifically against FB nonproducers, *F. verticillioides* is more resistant to externally added FB_1_ (38).

Despite all the functional analyses of *FUM* cluster genes for over two decades, the function of *FUM15* and *FUM16* has remained unclear so far. Also, the direct effect of *FUM15-FUM18* on ceramide biosynthesis has not yet been elucidated. Fum15 is a P450 monooxygenase, while Fum16 displays structural similarities with long-fatty-acid-CoA ligases (12). Our hypothesis was that they, too, may play a role in self-protection.

In this work, we uncovered the involvement of Fum15 and Fum16 in self-protection against FB_1_ in *F. verticillioides*, and additionally, in the budding yeast *Saccharomyces cerevisiae* as a heterologous host. Using an *in vitro* enzyme assay, we showed that Fum16 is a functional palmitoyl-CoA ligase, while the presence of the P450 monooxygenase Fum15 reduces extracellular levels of FB_1_.

## RESULTS

### Fum15 and Fum16 are co-compartmentalized with ceramide biosynthesis

**FIG 2:**
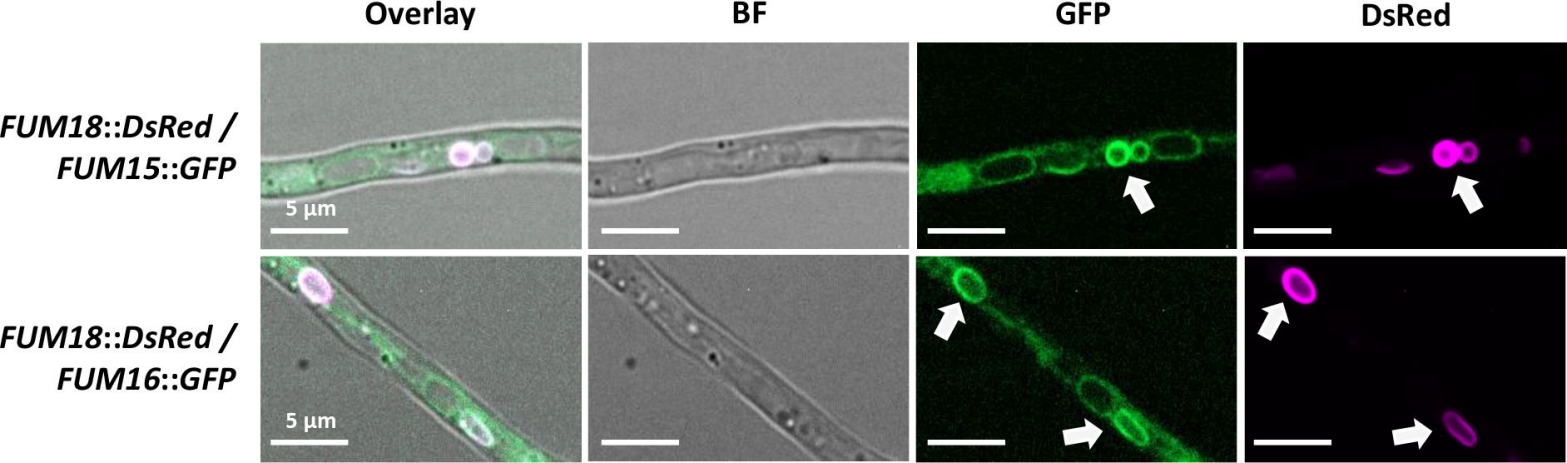
Co-localization of Fum15-GFP and Fum16-GFP with Fum18-DsRed. Shown are separate channels: brightfield (BF), GFP, DsRed, and the overlay. White arrows point to the perinuclear ER. Conidia were inoculated in ICI/6 mM Gln and grown as a standing culture overnight.

We started by performing localization studies of Fum15 and Fum16 by confocal microscopy. Both proteins were C-terminally tagged with a green fluorescent protein (GFP) and expressed from the same constitutive overexpression promoter (*Aspergillus nidulans PoliC*). In our previous work, we showed that the CerS Fum18 localizes to the ER and ER-derived vesicles (8). We thus used our mutant producing Fum18 C-terminally tagged with red fluorescent protein (DsRed) as a recipient strain. We made sure that the fluorescent proteins did not give signals when excited with inappropriate wavelengths (Fig. S2). Fum15-GFP and Fum16-GFP both showed co-localization with Fum18-DsRed in the perinuclear ER (Fig. 2).

### Establishment of an inducible *FUM21* overexpression strain

Although high enough concentrations of FB_1_ have been reported to have a negative impact on fungal growth, the *F. verticillioides* M-3125 wild type (WT) displays resistance to the toxin (8). For this reason, we designed a strain for inducible overproduction of FB_1_, by overexpressing the fumonisin BGC transcription factor gene *FUM21*. This allowed us to investigate the toxic effects on growth, ceramide biosynthesis, as well as the hypothesized contribution of *FUM15* and *FUM16* in self-protection. We employed the *tet^ON^* promoter system, which was successfully used in other fungal species such as *Aspergillus niger* (39) and *Fusarium fujikuroi* (40), for inducible, controllable production of metabolites by the addition of the inducer molecule doxycycline hyclate (doxy). The native promoter of the transcription factor gene *FUM21* was thus exchanged with the *tet^ON^* sequence.

**FIG 3:**
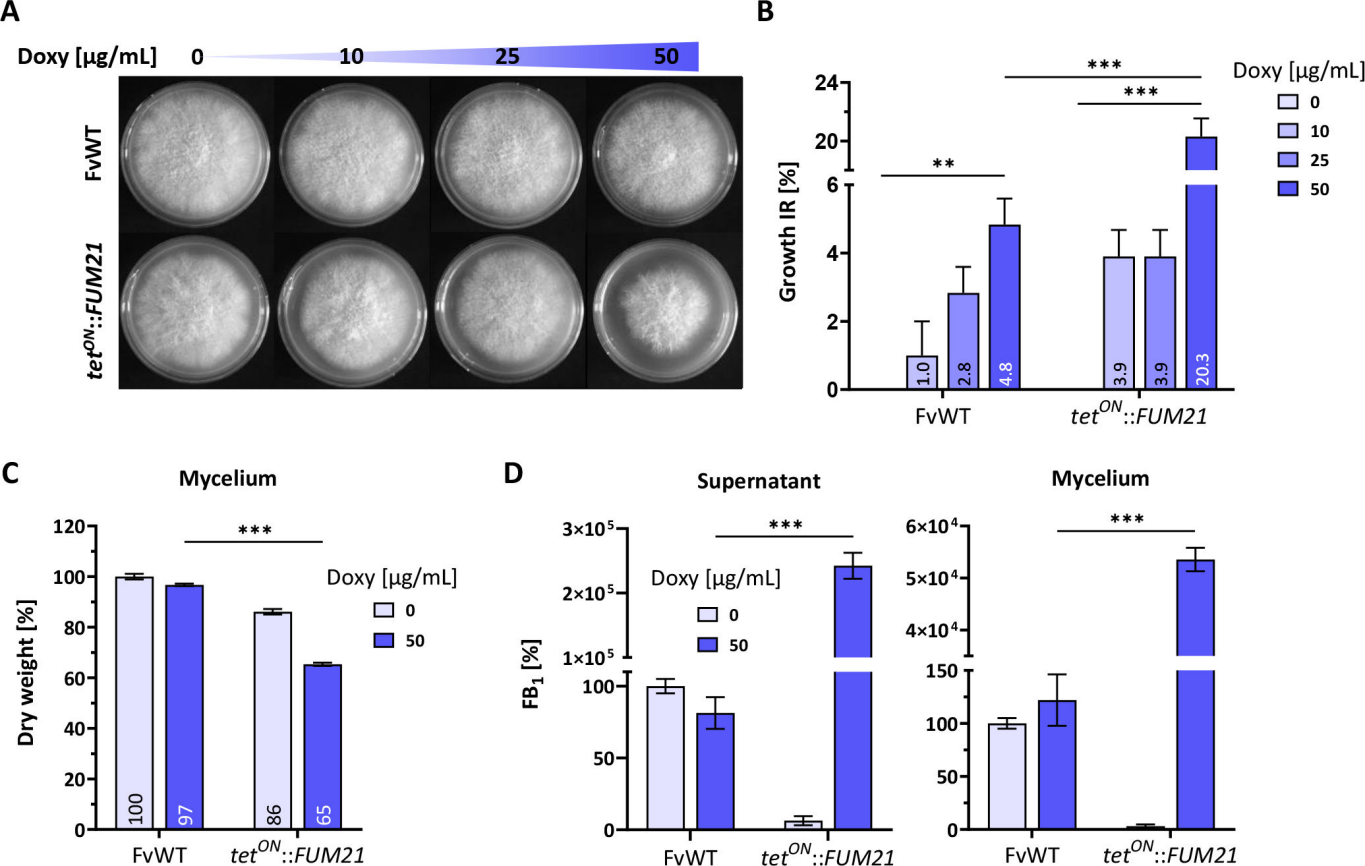
Analysis of FB_1_ production and phenotype of *F. verticillioides tet^ON^::FUM21*. (**A**) Growth assay performed on small complete medium plates and increasing concentration of doxycycline (doxy). The increased concentration of doxy results in the augmented expression of the TF *FUM21* and consequent increase of the *FUM* cluster activation. (**B**) The average diameter of the colonies was measured after 5 days with ±SD (*n* = 3). The growth inhibition rate (IR) is relative to the noninduced condition for each strain. (**C**) Biomass measurements of lyophilized mycelia, after 5 days of growth after induction in liquid culture (ICI/6 mM Gln). Dry weight of the WT at 0 µg/mL doxy was arbitrarily set to 100%. (**D**) Relative FB_1_ production of the WT and *tet^ON^::FUM21*, after 5 days of growth after induction in liquid culture (ICI/6 mM Gln). Shown are mean values with ±SD (*n* = 3). FB_1_ levels were normalized against an internal standard and related to the dry weight of the cultures. Production of the WT at 0 µg/mL doxy was arbitrarily set to 100%. Student’s *t*-test was used to assess statistical significance as indicated (**P < 0.01; ***P < 0.001).

In order to choose the most suitable doxy concentration to use in further assays, we first performed a growth inhibition assay to determine the effect of doxy concentration on the WT and overproduction strain *tet^ON^::FUM21* (Fig. 3A through C). Subsequently, we relatively quantified FB_1_ in the supernatant and extracted mycelium of liquid cultures with and without induction (Fig. 3D). The addition of the inducer appeared to have a toxic effect by itself as even the WT growth was affected by high concentrations of doxy. A maximum of 50 µg/mL doxy was used, which impaired WT growth by 4.8% on plate and 3.3% in liquid culture. In contrast to that, *tet^ON^::FUM21* was inhibited by the addition of 50 µg/mL doxy by more than 20%, both on plate and in liquid culture (Fig. 3B and C). We hypothesized that this more severe phenotype of *tet^ON^::FUM21* was predominantly caused by an increased FB_1_ production. Indeed, relative quantification of FB_1_ via high-performance liquid chromatography coupled to high-resolution mass spectrometry (HPLC-HRMS) revealed that the addition of 50 µg/mL doxy resulted in a drastic overproduction of FB_1_ in *tet^ON^::FUM21*, compared with the WT, both in the supernatant and mycelium (Fig. 3D). The identity of the chromatographic peak corresponding to FB_1_ was verified by comparison with an FB_1_ reference compound (Fig. S3A). Absolute quantification based on an FB_1_ standard curve (Fig. S3B) revealed an accumulation of 6.94 ± 0.03 µg/mL in the supernatant of the WT and of 671.74 ± 54.58 µg/mL in the supernatant of *tet^ON^::FUM21*. In the absence of doxy, FB_1_ levels in the overproduction strain were negligible, and no expression was detected of either *FUM21* or the biosynthetic gene *FUM8* (Fig. S4A), suggesting limited leakiness of the promoter, so that this strain was suitable for our analysis (Fig. 3D).

### Fum15 and Fum16 are involved in self-protection against FB_1_

In order to further investigate the function and significance of *FUM15* and *FUM16*, single-deletion mutants in the overexpression strain were generated (*tet^ON^::FUM21*/Δ*fum15* and *tet^ON^::FUM21*/Δ*fum16*). As a negative control, *tet^ON^::FUM21*/Δ*fum8* was also generated. The biosynthetic enzyme Fum8 is an aminotransferase that performs the second catalytic step in fumonisin biosynthesis; it catalyzes the condensation of l-alanine with the octadecanoic acid precursor. Thereby, deletion of *FUM8* abolishes fumonisin production in our overexpression strain (see FB_1_ quantification below). Furthermore, the CerS mutant *tet^ON^::FUM21*/Δ*fum18* was generated as a positive control based on previously published results (8), but also to expand on the self-defense role of *FUM18* as a CerS homolog. Finally, a strain was also generated, in which the whole non-biosynthetic subcluster, *FUM15* to *FUM19*, was deleted (*tet^ON^::FUM21*/Δ*fum15-19*).

**FIG 4:**
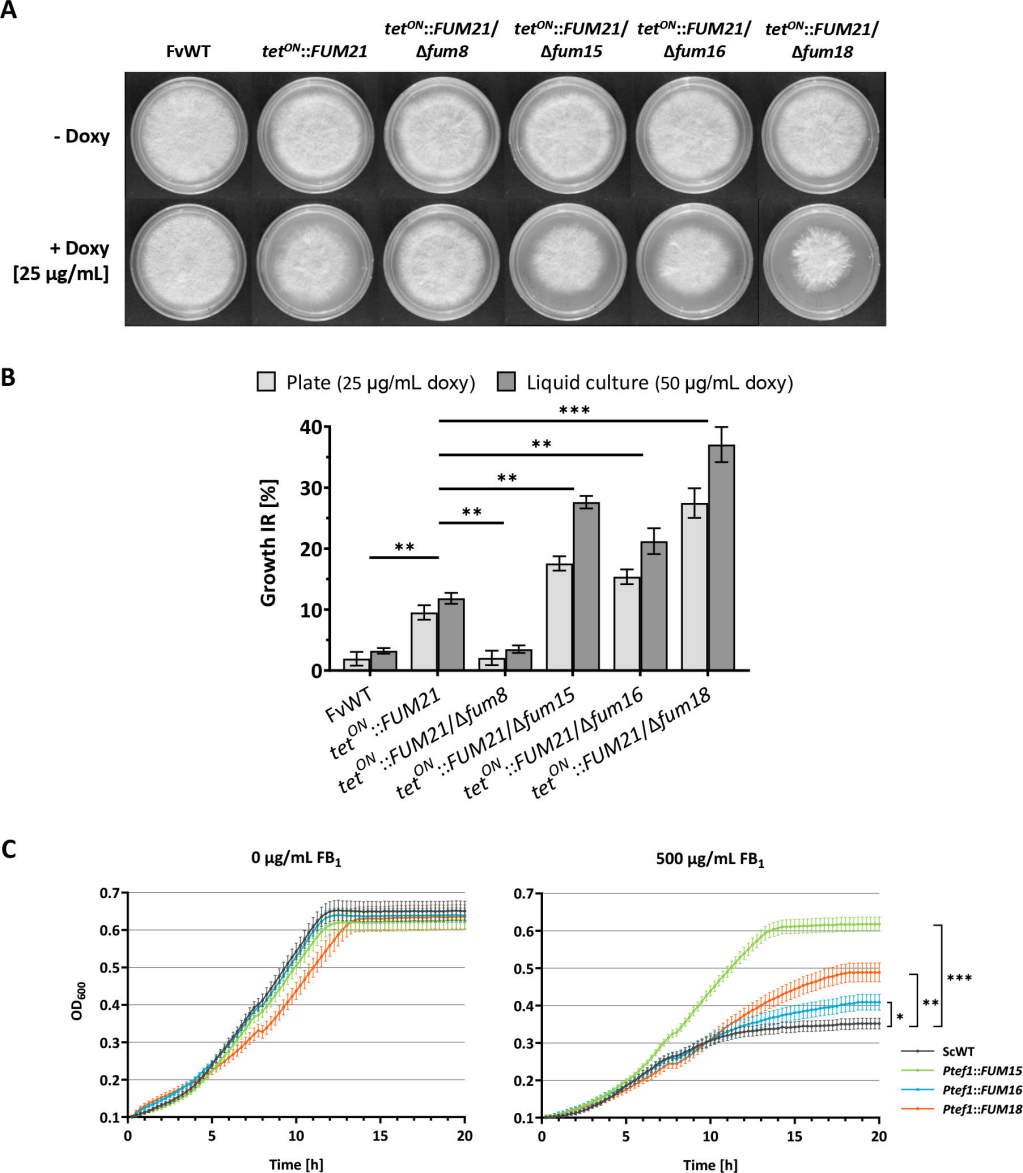
Growth phenotype analysis of the deletion mutants in *F. verticillioides* and of *S. cerevisiae* expressing *FUM15-18*. (**A**) Growth inhibition of *F. verticillioides* WT, *tet^ON^::FUM21* and deletion strains of the latter. The plate assay was performed on complete medium plates under uninduced and induced (50 µg/mL doxy) conditions. (**B**) The growth inhibition rate (IR) is relative to the noninduced condition for each strain. The average diameter of the colonies was measured after 6 days. Biomass of lyophilized mycelia was quantified after 5 days of growth after induction in liquid culture (ICI/6 mM Gln). Data are mean values ± SD (*n* = 3). (**C**) Growth curves of *S. cerevisiae* expressing *FUM15*, *FUM16*, or *FUM18* over 20 h in SD-Ura, either in the absence or presence of 500 µg/mL FB_1_ (mean ± SD, *n* = 3). The blank was subtracted from each data point, and the curves were adjusted to an initial OD_600_ of 0.1. Statistical analysis was performed on the OD value at 20 h. Student’s *t*-test was used to assess statistical significance as indicated (*P < 0.05; **P < 0.01; ***P < 0.001).

These deletion mutants were tested both in plate assays and in liquid cultures, under uninduced and induced conditions, to evaluate their growth phenotype (Fig. 4A and B). A concentration of 50 µg/mL doxy was used in this study for FB_1_ overproduction in liquid cultures, while 25 µg/mL was instead used in plate assays as we observed that our strains were more susceptible to FB_1_ on solid medium (Fig. S5). Overproduction of FB_1_ resulted in irregularly shaped hyphal growth at the colony periphery of *tet^ON^::FUM21* mutants, with lack of aerial hyphae, compared with the uninduced strains (Fig. 4A). Doxy induction caused statistically significant mycelial growth inhibition after 6 days on solid medium and after 5 days in induced liquid cultures compared with the equally treated WT, with the exception of the FB_1_-nonproducer *tet^ON^::FUM21*/Δ*fum8*. All three non-biosynthetic single-gene deletions (Δ*fum15*, Δ*fum16*, and Δ*fum18* deletion in the *tet^ON^::FUM21* background) caused a higher growth inhibition rate compared with *tet^ON^::FUM21* alone (Fig. 4B). Among those, deletion of *FUM18* caused the most severe effect among the three, with a growth inhibition rate of 28% on solid medium and 38% in liquid culture, while deletion of *FUM16* caused the least severe effect (15% and 21%, respectively) (Fig. 4B). The complete subcluster deletion of *FUM15-19* did not result in an additional growth inhibition compared with *tet^ON^::FUM21*/Δ*fum18* (Fig. S5).

Expression analysis of the *F. verticillioides* WT, *tet^ON^::FUM21* and deletion mutants thereof revealed that single deletion of *FUM15*, *FUM16,* or *FUM18* did not result in a compensatory upregulation of the adjacent genes (Fig. S4A). Deletion of *FUM8* in the *tet^ON^::FUM21* background, abolishing FB_1_ biosynthesis, failed to fully induce expression of *FUM18* and *FUM19*, which are driven by a bidirectional promoter (Fig. 1A). We previously reported that FB_1_ feeding triggered *FUM18* and *FUM19* expression (8), and now additionally report an induction of *FUM15* and *FUM16* in the WT by 10 µg/mL exogenously added FB_1_ (Fig. S4B). We noted that the expression of *FUM15* was not abolished in *tet^ON^::FUM21* in the absence of the inducer doxy, suggesting that *FUM15* is not fully under the control of the cluster-specific transcription factor (Fig. S4A). This was corroborated by analysis of the Δ*fum21* deletion mutant (8), which showed ca. 20% of the *FUM15* expression detected in the WT background (Fig. S4B). These expression data are in line with the idea that Fum15 and Fum16, in addition to Fum18, could play a protective role.

To confirm the direct involvement of Fum15 and Fum16 in self-protection against FB_1_, we designed a heterologous expression experiment with *S. cerevisiae. S. cerevisiae* strains were engineered to constitutively express *FUM15* (*Ptef1::FUM15*), *FUM16* (*Ptef1::FUM16*), or *FUM18* (*Ptef1::FUM18*) (8). We incubated these strains in the absence or presence of 500 µg/mL FB_1_ and monitored the optical density at 600 nm (OD_600_) over a period of 20 h (Fig. 4C). The incubation with FB_1_ impaired *S. cerevisiae* WT growth by 47%. Expression of *FUM16* and *FUM18* resulted in a partial rescue as growth was impaired by only 36% and 23%, respectively. Surprisingly, *S. cerevisiae* expressing *FUM15* displayed almost no growth inhibition compared with the control condition and could reach higher OD_600_ compared with the other mutants in the presence of the toxin (Fig. 4C). Expression analysis showed that all three genes were expressed in a similar range (ca. 2.5- to 3.5-fold relative to the housekeeping gene actin), excluding aberrant expression as a reason for the lower performance of *FUM16* and *FUM18* in the heterologous system (Fig. S4C).

Taken together, these results demonstrate that the enzymes Fum15 and Fum16 by themselves play a role in self-protection against FB_1_, both in homologous and heterologous hosts.

### Deletion of either *FUM15*, *FUM16*, or *FUM18* affects the abundance of ceramide intermediates

**FIG 5:**
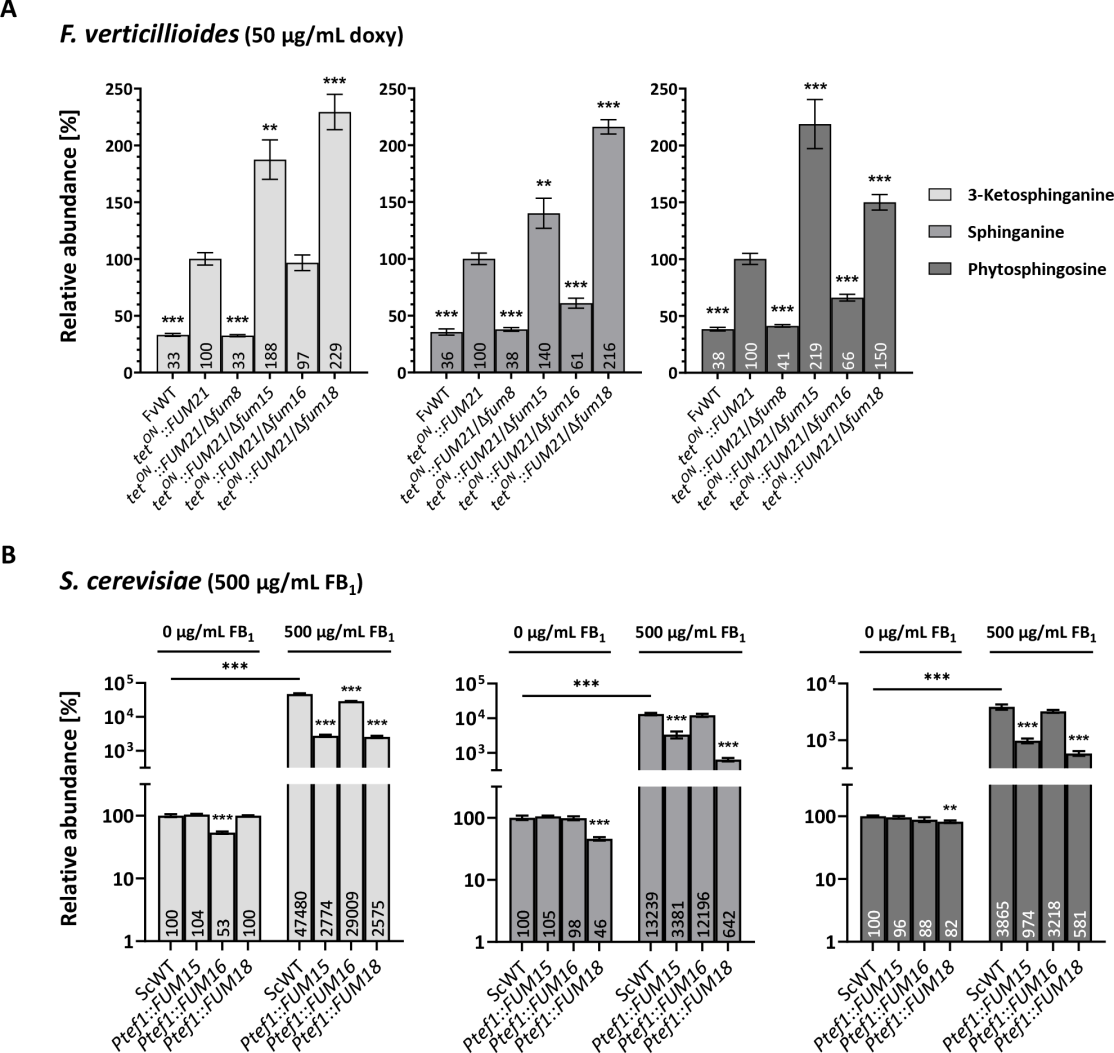
Analysis of the production of ceramide intermediates. (**A**) Relative abundance of three ceramide intermediates, 3-ketosphinganine, sphinganine, and phytosphingosine, extracted from the mycelium of the *F. verticillioides* WT, *tet^ON^::FUM21* and the deletion mutants Δ*fum8*, Δ*fum15*, Δ*fum16*, Δ*fum18* in the *tet^ON^::FUM21* background. All cultures were induced with 50 µg/mL doxy for 5 days in liquid culture (ICI/6 mM Gln), and the production of *tet^ON^::FUM21* was set to 100% (mean ± SD, *n* = 3). (**B**) Relative abundance of the abovementioned intermediates, extracted from the cell pellet of the *S. cerevisiae* WT (carrying the empty plasmid pYES::*Ptef1*), *Ptef1::FUM15*, *FUM16*, and *FUM18* grown in SD-Ura. The abundance in the nontreated WT was set to 100%, and statistical analysis was performed within each condition (0 and 500 µg/mL FB_1_) if not specified otherwise (horizontal line between conditions). Shown are mean values ± SD (*n* = 3). Student’s *t*-test was used to assess statistical significance (**P < 0.01; ***P < 0.001).

Fum18 was already demonstrated to contribute to resistance against FB_1_ by functioning as a CerS on its own and thus providing supplementary CerS activity to the ceramide biosynthetic pathway (8). We asked whether Fum15 (a predicted P450 monooxygenase) or Fum16 (a predicted long-fatty-acid-CoA ligase) could also confer self-protection against the toxin similarly, supplementing the sphingolipid biosynthetic pathway. We performed an HPLC-HRMS analysis of the extracted mycelium of *tet^ON^::FUM21*, as well as Δ*fum8*, Δ*fum15*, Δ*fum16*, and Δ*fum18* deletions in the *tet^ON^::FUM21* background, grown in doxy-induced liquid cultures. We measured the abundance of 3-ketosphinganine, sphinganine, and phytosphingosine (Fig. 5A). Overproduction of FB_1_ by *tet^ON^::FUM21* blocked ceramide biosynthesis and caused a statistically significant increase in the precursors 3-ketosphinganine (3-fold), sphinganine (2.7-fold), and phytosphingosine (2.5-fold) compared with the induced WT (Fig. 1B). The increase was even more severe when either *FUM15* or *FUM18* were deleted, for all three intermediates (Fig. 5A), apparently causing a more severe inhibition of ceramide biosynthesis. These results confirmed the role of Fum18 as a CerS (8) but also provided new insight into the biosynthetic steps that are supplemented by the enzyme, which is thus acting on both of its direct substrates sphinganine and phytosphingosine (Fig. 1B). *FUM16* deletion resulted in a ca. 40% decrease of sphinganine and phytosphingosine (Fig. 5A), indicating that Fum16 may have a catalytic role upstream of ceramide biosynthesis.

### Fum16 is directly involved in ceramide intermediate biosynthesis, while Fum15 is not

In order to understand if Fum15 and Fum16 affect ceramide intermediates directly, we analyzed 3-ketosphinganine, sphinganine, and phytosphingosine abundance in *S. cerevisiae* strains expressing *Ptef1::FUM15*, *Ptef1::FUM16*, and *Ptef1::FUM18* in the absence and presence of 500 µg/mL FB_1_ (Fig. 5B). In the absence of the toxin, Fum15 was the only enzyme that did not lead to a change in ceramide intermediate abundance compared with the *S. cerevisiae* WT. In contrast, Fum16 activity by itself was responsible for a decrease in 3-ketosphinganine of 47%, which was the earliest tested intermediate (Fig. 1B and 5B). As expected, Fum18 caused a decrease in its direct precursors sphinganine and phytosphingosine of 54% and 18%, respectively (Fig. 5B). The ratio of sphinganine-to-phytosphingosine in *Ptef1::FUM18* can be explained by sphinganine being both a substrate for dihydroceramide synthesis and a precursor of phytosphingosine, which in turn is a substrate for phytoceramide synthesis (Fig. 1B). When exposed to the toxin, both *FUM15*- and *FUM18*-expressing strains displayed a drastic decrease in all three intermediates compared with the WT (Fig. 5B), a phenotype that was opposite of the one observed for the *F. verticillioides* deletion mutants *tet^ON^::FUM21*/Δ*fum15* and *tet^ON^::FUM21*/Δ*fum18* (Fig. 5A).

From these results, it appears that although Fum15 has an effect on the equilibrium of ceramide biosynthesis, and counteracts CerS inhibition by FB_1_, it does not do so by directly supplementing the pathway. On the contrary, evidence was gained that Fum16—and Fum18, as expected—has a direct impact on ceramide biosynthesis.

### Fum15 affects FB_1_ levels in *F. verticillioides*, thus alleviating self-toxicity

**FIG 6:**
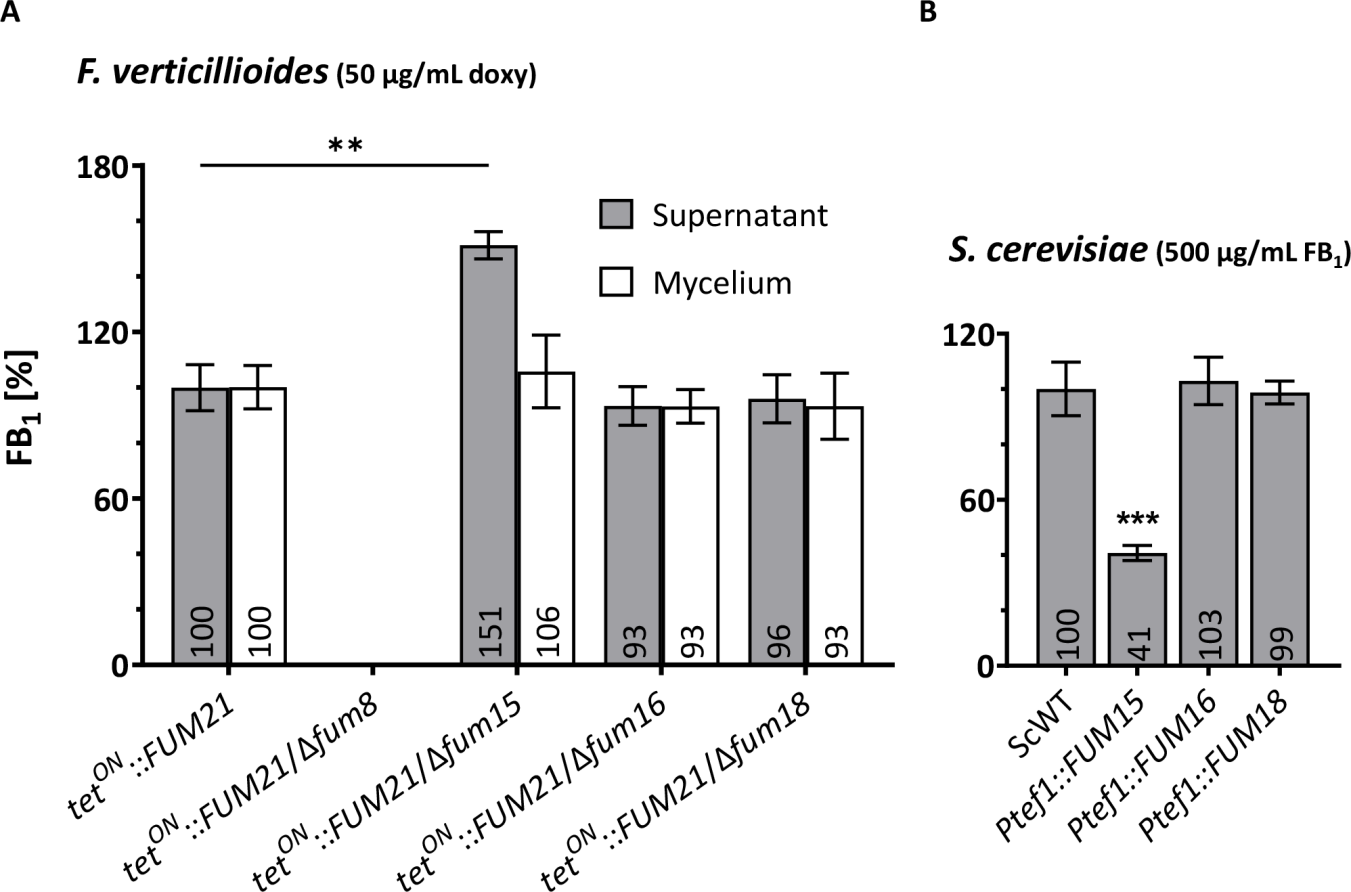
Analysis of the production of FB_1_. (**A**) Relative abundance of FB_1_ in the supernatant and extracted mycelium of the *F. verticillioides* WT, *tet^ON^::FUM21,* and the deletion mutants Δ*fum8*, Δ*fum15*, Δ*fum16*, Δ*fum18* in the *tet^ON^::FUM21* background. All cultures were induced with 50 µg/mL doxy for 5 days in liquid culture (ICI/6 mM Gln), and the production of *tet^ON^::FUM21* was set to 100% (mean ± SD, *n* = 3). (**B**) Relative abundance of FB_1_ in the supernatant of the *S. cerevisiae* WT (carrying the empty plasmid pYES::*Ptef1*), *Ptef1::FUM15*, *FUM16*, and *FUM18*, grown in SD-Ura and treated with 500 µg/mL FB_1_. Abundance in the WT was set to 100%. Shown are mean values ± SD (*n* = 3). Student’s *t*-test was used to assess statistical significance compared with the control or as indicated (**P < 0.01; ***P < 0.001).

In the previous experiments, we observed that Fum15 performs a function that is beneficial for survival when exposed to FB_1_, both in *F. verticillioides* and *S. cerevisiae* (Fig. 4). We also discovered that the catalytic activity of Fum15 has an indirect effect on ceramide biosynthesis (Fig. 5). We thus raised the question whether Fum15 could possess an activity toward fumonisin itself. We analyzed the phenotype of the *F. verticillioides* single-deletion mutants in regard to FB_1_ production under inducing conditions and in comparison to the genetic background of *tet^ON^::FUM21* (Fig. 6A). As a control, we also performed the analysis on the *S. cerevisiae* overexpression strains treated with FB_1_ (Fig. 6B). FB_1_ production was quantified in the supernatant and mycelium of *F. verticillioides* liquid cultures by HPLC-HRMS. Deletion of either *FUM16* or *FUM18* in the *tet^ON^::FUM21* background had no impact on FB_1_ production (Fig. 6A). This was in accordance with previous research (8, 41). However, deletion of *FUM15* resulted in a statistically significant increase of the toxin by 50% in the supernatant, compared with the background strain *tet^ON^::FUM21*, but no change was detected in the mycelium (Fig. 6A). When analyzed in *S. cerevisiae*, the expression of *FUM16* or *FUM18* did not lead to a change in FB_1_ abundance in the supernatant. Expression of *FUM15*, however, led to a decrease of 60% compared with the treated *S. cerevisiae* WT (Fig. 6B). These results are in accordance with the change in all three quantified ceramide intermediates described above. The increase and decrease of FB_1_ in the used *F. verticillioides* and *S. cerevisiae FUM15* strains, respectively, explain the stronger and weaker inhibition of ceramide biosynthesis in these organisms, and explain why they show the same trend as the CerS-encoding *FUM18* deletion and overexpression strains (Fig. 5). Our data point toward the fact that the P450 monooxygenase Fum15 could chemically modify FB_1_ and thereby detoxify it. No new peak(s) could be detected under our cultivation conditions, neither in *S. cerevisiae* nor in *F. verticillioides*. An explanation in agreement with these observations is that the resulting metabolite could be unstable and quickly degraded.

### Fum16 is a functional palmitoyl-CoA ligase

**FIG 7:**
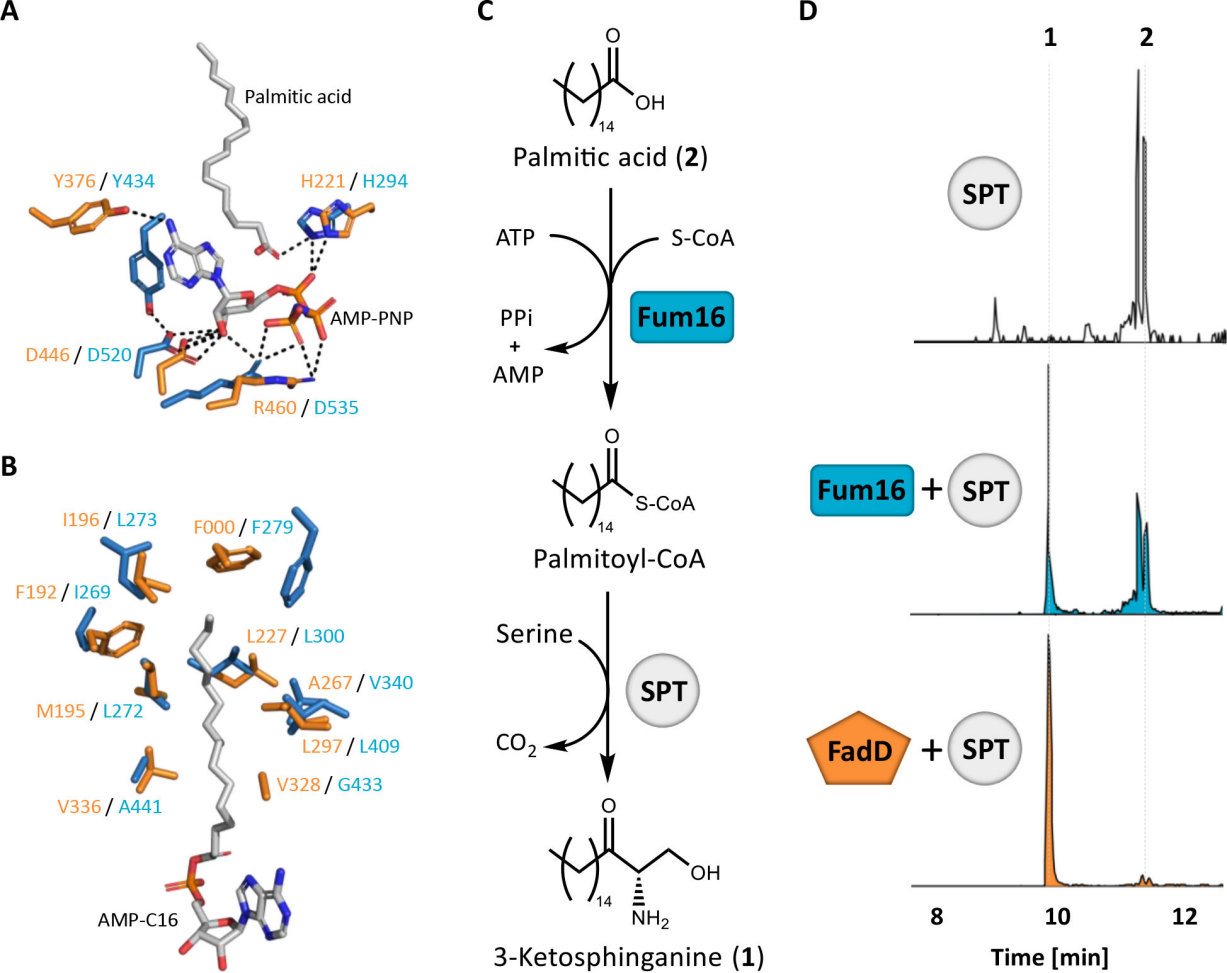
*In vitro* activity assay of Fum16 for fatty-acid-CoA ligase activity. (**A and B**) Stereo representations of two FadD23 complexes (orange), aligned with the active site of the Fum16 structure predicted by AlphaFold2 (cyan). (**A**) Hydrogen bonds (black dotted lines) in the active site of the AMP-PNP-FadD23 complex. AMP-PNP and palmitic acid are shown in gray. (**B**) The hydrophobic AMP-C16-FadD23 complex. (**C**) Graphical representation of the reaction catalyzed by Fum16, namely the ligation of CoA with palmitic acid. (**D**) Shown are the total ion chromatograms of the HPLC-HRMS measurements of the assay. When Fum16 was present in the reaction, a peak corresponding to 3-ketosphinganine (1) appeared (cyan chromatogram). The negative control contained only the SPT enzyme from *S. paucimobilis* (white chromatogram), while the positive control contained SPT with the bacterial CoA ligase FadD (orange chromatogram).

Fum16 is predicted to be a long-fatty-acid-CoA ligase, based on the highest BLASTX score for its open-reading frame (12). In our investigation, we further pinpointed Fum16 structure and function using the web-based tool Protein Homology/analogY Recognition Engine V 2.0 (Phyre2) (42). A total of 601 residues (89% coverage) of Fum16 were modeled with a 100% confidence against several fatty-acyl-CoA ligases, spanning several domains of life: from the plant 4-coumaric-acid-CoA ligase from *Populus tomentosa* (Pt4CL) (43) to the bacterial fatty-acyl-AMP ligase from *Mycobacterium tuberculosis* (FadD23) (44). As a verification, we aligned the Phyre2 prediction with a Fum16 structure obtained via the artificial intelligence program AlphaFold2 (Fum16-AF2) (45). The alignment yielded a root mean square deviation (RMSD) of 1.840, which proved the similarity of both models (Fig. S6A and B). The alignment of Fum16-AF2 with FadD23, complexed with palmitic acid and an ATP analog, revealed an impressive residue conservation at the active site in regard to both amino acid composition and hydrogen bond interactions with the substrates (Fig. 7A). Furthermore, Fum16-AF2 also appeared to possess an N-terminal hydrophobic pocket suitable for accommodating palmitic acid or an hexadecanoyl adenylate intermediate (Fig. 7B).

Based on our predictions, we next aimed to prove Fum16’s activity in ceramide intermediate biosynthesis, which was expected to be the ligation of coenzyme A (CoA) with palmitic acid to yield palmitoyl-CoA. We thus established an *in vitro* assay. Fum16 was rendered soluble for bacterial expression and purification by truncating its predicted N-terminal transmembrane domain and N-terminally fusing it to a maltose-binding protein (Fig. S6C through F). Palmitic acid was supplied in the reaction and Fum16 was indeed able to synthesize palmitoyl-CoA, which in turn could be condensed with serine by a serine palmitoyl transferase (SPT, from *Sphingomonas paucimobilis*) to yield the final product 3-ketosphinganine. The consumption of palmitic acid and the production of 3-ketosphinganine were detected by HPLC-HRMS (Fig. 7C and D). A purified fatty-acid-CoA ligase from *Escherichia coli* (FadD) was used as a positive control. The negative control only included SPT with the substrates (Fig. 7D).

Thus, we demonstrated the function of Fum16 as a palmitoyl-CoA ligase. This finding is in accordance with what was described above for the ceramide intermediate analysis (Fig. 5) and unequivocally provides proof that Fum16 contributes to self-protection against FB_1_ as functional palmitoyl-CoA ligase (Fig. 1B).

## DISCUSSION

Mycotoxin-producing organisms evolved several strategies to overcome self-toxicity, like the duplication of target enzymes, efficient toxin secretion mechanisms, enzymatic detoxification, and pathway compartmentalization (6–9). In our previous work, we identified the function of three genes in the *FUM* cluster of *F. verticillioides* that are located in a subcluster relevant for self-protection, *FUM17*, *FUM18*, and *FUM19* (8). In the present study, we expanded on this knowledge by postulating the role of two additional genes, *FUM15* (P450 monooxygenase) and *FUM16* (palmitoyl-CoA ligase), and discovered that they, too, carry out self-protective functions (Fig. 1B). We are therefore presenting evidence for the existence of a five-gene subcluster *FUM15-19* specifically dedicated to self-protection against the sphingolipid biosynthesis inhibitor FB_1_ in *F. verticillioides*.

The production of fungal SMs is tightly controlled not only on a temporal level but also spatially (46). In our previous work, we showed that the CerS homologs Fum17 and Fum18 are compartmentalized and co-localize with ceramide biosynthesis in the ER, while the final biosynthetic step to FB_1_ by Fum3 is performed in the cytosol (8). Here, we demonstrated an equivalent localization for Fum15 and Fum16 in the ER. While certain P450 monooxygenases have been observed in various cellular compartments, they are typically located in the ER (47) where they associate with NADPH-cytochrome P450 reductases for electron supply (48). In contrast to that, long-fatty-acid-CoA ligases are predominantly found in peroxisomes (49). The localization of Fum16 to the ER thus offered an initial indication of its role in ceramide biosynthesis.

In this work, the involvement of *FUM15* and *FUM16* in protection against FB_1_ was demonstrated both in the native host *F. verticillioides* and in the heterologous host *S. cerevisiae*. Deletions of either *FUM15*, *FUM16*, or *FUM18* in an FB_1_-overproducing strain of *F. verticillioides* resulted in growth inhibition, both on solid medium and in liquid culture. On the opposite, constitutive heterologous expression of these genes in *S. cerevisiae* conferred protection against FB_1_. *FUM18* appears to be the non-biosynthetic gene conferring the highest degree of protection in the *F. verticillioides* experimental setup. This was confirmed by observing no difference in the growth inhibition phenotype both when deleting *FUM18* by itself and in combination with the other non-biosynthetic genes *FUM15-19*. In contrast, in the *S. cerevisiae* system, it was *FUM15* expression that resulted in the highest survival, with a growth phenotype almost comparable to the untreated *S. cerevisiae* WT. While all three genes were expressed to similar strength from the overexpression promoter, we cannot exclude differences in the resulting protein levels as we did not perform codon optimization. Alternatively, it might be due to variations in enzyme activities between the different host organisms.

Subsequently, we observed that the deletion of either *FUM15*, *FUM16*, or *FUM18* affects ceramide homeostasis in *F. verticillioides*. In *S. cerevisiae*, in the absence of FB_1_, the expression of *FUM15* did not alter the levels of ceramide intermediates, which suggests that it does not have a direct enzymatic function in ceramide biosynthesis. Conversely, *FUM16* expression in untreated *S. cerevisiae* cultures was sufficient to influence ceramide intermediate biosynthesis. There was no effect of *FUM16* expression on FB_1_ levels in *S. cerevisiae*, which was consistent with findings for the *FUM16* deletion mutant of *F. verticillioides* (this study; 41), and supported the hypothesis that it plays a direct role in supplementing the ceramide pathway. This could be unequivocally verified by an *in vitro* enzymatic assay, where we demonstrated that Fum16 is a functional palmitoyl-CoA ligase (Fig. 1B). Recently, we described a similar case of enzymatic supplementation of sphingolipid biosynthesis serving as a self-protection mechanism in *Aspergillus*. We explored the interactions between sphingolipid metabolism and sphingofungin biosynthesis in *Aspergillus fumigatus*, with the latter being a sphingolipid biosynthesis inhibitor acting on SPT (37). We demonstrated that the biosynthetic enzyme SphA (aminotransferase) plays a dual role in both sphingofungin and sphingolipid biosynthesis as it performs SPT activity in the presence of the serine and palmitoyl-CoA precursors (50).

From an evolutionary perspective, *FUM15* seems to play an important role. The fumonisin BGC exhibits significant variation among black aspergilli (51), which are reported to primarily produce the FB analogs FB_2_ and FB_4_ (precursor of FB_2_), but not FB_1_ (52). Among the non-biosynthetic genes identified for self-protection in *Fusarium* (*FUM15-19*), only *FUM15* is present in the *FUM* cluster of both fumonisin-producing and nonproducing strains of *A. niger* (53). Furthermore, in nonproducing isolates of *Aspergillus luchuensis*, *Aspergillus brasiliensis*, and *Aspergillus tubingensis*, the partial *FUM* cluster exclusively retained *FUM1* and *FUM15* (51). Our expression data revealed that *FUM15* was not under the tight control of the transcription factor Fum21, similar to what was reported for *A. niger* previously (54).

Our data do not provide evidence for a direct role of Fum15 in ceramide biosynthesis. Instead, we suggest that it chemically modifies and thereby detoxifies FB_1_ due to the fact that *FUM15* deletion in *F. verticillioides* resulted in increased extracellular FB_1_ levels, while the opposite was observed for the heterologous expression of *FUM15* in FB_1_-supplemented *S. cerevisiae*. P450 monooxygenases have been largely reported to be involved in the detoxification of mycotoxins through biotransformation and chemical modification (55). In *F. fujikuroi*, P450 monooxygenase(s) were shown to be involved in the detoxification of fusaric acid (56) and gibepyrone A (40). Other examples are the hydroxylation and epoxidation of aflatoxin B_1_ (7, 55), as well as the hydroxylation of deoxynivalenol (7). It is apparent that other cluster-independent mechanisms cooperate to confer protection against self-toxicity by FB_1_ in *F. verticillioides* since the deletion of the complete subcluster *FUM15-19* still displayed viability. These are likely cluster-independent ABC transporters compensating for the loss of *FUM19* (8). Additionally, a gene directly adjacent to the *FUM* cluster, *FvZBD1*, is postulated to act as a genetic repressor of fumonisin production (57).

Based on the results of this study, we conclude that the non-biosynthetic genes, *FUM15-19*, in the *F. verticillioides FUM* cluster compose a subcluster of contiguous genes, which are able to confer self-resistance against the toxic effects of FB_1_. *FUM16*, *FUM17*, and *FUM18* do so by supplementing ceramide biosynthesis as palmitoyl-CoA ligase and CerSs, respectively (this study; 8)). *FUM19* encodes an ABC transporter that acts as a repressor of the *FUM* gene cluster (8), while *FUM15* is possibly involved in chemical detoxification of FB_1_. Addressing mycotoxin contamination is crucial for maintaining a safe and sustainable food supply, highlighting the importance of continued research and monitoring (1). In particular, future identification of these self-protection subclusters could uncover mycotoxins with currently unknown biological functions and potentially assist in developing plant cultivars with enhanced resistance.

## MATERIALS AND METHODS

### General molecular methods

For amplification of desired DNA fragments from template DNA, the Phusion Flash High-Fidelity PCR Master Mix (Thermo Fisher Scientific, Dreieich, Germany) was used. All primers used for PCR amplification, diagnostic PCR, and Southern blots are listed in Table S1. For DNA enzymatic digestions, restriction enzymes by New England Biolabs (Frankfurt am Main, Germany) were used. DNA extraction from agarose gel was performed using the GeneJET Gel Extraction Kit (Thermo Fisher Scientific, Dreieich, Germany).

Vector assembly was achieved using either *S. cerevisiae* transformation-associated recombination (TAR) (58) or Gibson assembly (59) and checked with restriction digestion and sequencing. Plasmid DNA purification was performed using the NucleoSpin Plasmid Mini Kit (Macherey-Nagel, Düren, Germany).

### Plasmid construction

Promoter exchange of *F. verticillioides FUM21* with the inducible *tet^ON^* promoter (Fig. S7) started out by amplifying upstream and (intragenic) downstream sequences (relative to the start codon) with the primer pairs fum21_5F/nat1_fum21_5R and TETon_FUM21_3F/TetON_FUM21_3R2, respectively. *tet^ON^* (39) was amplified from pYES2-ptrA-TetON (37) with nat1_tetON_F2/gpda_for_vv. *natR* was amplified from pZPnat1 (GenBank accession no. AY631958.1) with hph_trpC_F/nat1_R. The obtained fragments were cloned into the BamHI/HindIII-digested shuttle vector pYES2 (Life Technologies, Darmstadt, Germany) using Gibson assembly.

Deletion vectors harbored ~1 kb upstream and downstream flanks of the gene of interest, as well as a deletion cassette. Flanks were amplified using primer pairs 5F/5R and 3F/3R (Table S1), as well as *F. verticillioides* genomic DNA. *FUM8*, *FUM15*, *FUM16*, *FUM18*, and *FUM15-19* were deleted by exchange with the hygromycin B resistance gene under the control of *A. nidulans PgpdA* (Fig. S7). For that purpose, *hphR* was amplified from pUC-hph (60) with gpda_for_vv/Hph_Rev_VV2. The complete vectors were assembled under the use of a linearized (HindIII/XbaI) pYES2, applying TAR cloning.

Microscopy vectors were obtained by fusing the genes of interest to the constitutive *A. nidulans* promoter *PoliC* and C-terminal fusion to *GFP* (Fig. S8). To this end, the insert was cloned into NcoI-digested pNDH-OGG (61), applying TAR cloning.

*S. cerevisiae* expression vectors (Fig. S9) were constructed by amplifying the inserts from *F. verticillioides* cDNA. The inserts were then cloned into the amplified (primer pair TEF_Rv/pYes2_cyc1T_Fw) backbone pYES2::*Ptef1* (62) using Gibson assembly.

Finally, the bacterial overexpression vector was obtained by amplifying an N-terminally truncated version of *FUM16* from *F. verticillioides* cDNA. The insert was then cloned into the BamHI/HindIII-digested vector pMALC2HTEV (Addgene no. 75286) using Gibson assembly.

### *F. verticillioides* transformation and analysis of transformants

Protoplast transformation of *F. verticillioides* was carried out as described elsewhere (63). Here, 20–30 µg of the circular vector was transformed to achieve overexpression of GFP-tagged *FUM15* and *FUM16*, while PCR-amplified fragments were transformed for inducible expression of *FUM21*, and for gene deletions. Transformants of *tet^ON^::FUM21* were maintained on complete medium (CM) plates (64) containing 200 µg/mL nourseothricin (Jena Bioscience, Jena, Germany). This strain was then used as background for the single-gene deletions, and the transformants were grown on CM plates containing both hygromycin B (200 µg/mL, InvivoGen Europe, Toulouse, France) and nourseothricin (200 µg/mL). Homologous recombination of the flanks and absence of untransformed nuclei were tested by diagnostic PCR, while Southern blot experiments excluded additional ectopic integration events. Thus, 2–10 independent transformants were verified for *tet^ON^::FUM21*, *tet^ON^::FUM21*/Δ*fum8*, *tet^ON^::FUM21*/Δ*fum15*, *tet^ON^::FUM21*/Δ*fum16*, *tet^ON^::FUM21*/Δ*fum18*, and *tet^ON^::FUM21*/Δ*fum15-19* mutants (Fig. S7). The microscopy strain *FUM18::DsRed* (8) was maintained on CM plates containing 200 µg/mL nourseothricin and was used as background for the transformation of *FUM15::GFP* and *FUM16::GFP*. The transformants were grown on CM plates containing both hygromycin B (200 µg/mL) and nourseothricin (200 µg/mL). The ectopic integration of the plasmids was verified by diagnostic PCR (Fig. S8).

### *E. coli* media and growth conditions

For cloning, *E. coli* DH5α cells were incubated on Luria-Bertani (LB) plates supplemented with 60 µg/mL carbenicillin (Carl Roth, Karlsruhe, Germany) and incubated at 37°C overnight. In order to isolate plasmids, single colonies were inoculated in LB medium with carbenicillin and incubated at 37°C and 180 rpm overnight. For protein production, *E. coli* BL21 (DE3) competent cells were transformed with the appropriate plasmid, grown overnight (37°C, 180 rpm) in LB medium with kanamycin (25 µg/mL, Carl Roth, Karlsruhe, Germany).

### *S. cerevisiae* media and growth conditions

*S. cerevisiae* BY4741 (Euroscarf, Oberursel, Germany) was used as a background strain and is thus referred to as ScWT. General maintenance of the generated *S. cerevisiae* mutants was performed on solidified synthetic defined medium lacking uracil (SD-Ura). *S. cerevisiae* cells for plate assays were pre-cultured in liquid SD-Ura and shaken overnight in 100 mL flasks with baffles at 30°C and 180 rpm. All cultivations for growth curves, survival assays, as well as the analysis of ceramide intermediates were carried out in SD-Ura at 30°C. For gene expression analysis, 20 mL SD-Ura in 100 mL flasks with baffles was inoculated with a dense overnight culture to an OD_600_ of 0.2 and grown out to an OD_600_ of 1 before harvesting and freeze-drying.

### *F. verticillioides* media and growth conditions

*F. verticillioides* M-3125 (65) was used as parental FvWT strain for the analysis. General maintenance of fungal strains was performed on solidified CM, with or without appropriate selection (200 µg/mL nourseothricin, 200 µg/mL hygromycin B). For the cultivation of strains in liquid culture, 100 mL of Darken pre-culture (66) in 300 mL Erlenmeyer flasks was inoculated with a mycelial plug and shaken for 3 days at 180 rpm and 28°C. For the main culture, 500 µL of the pre-culture was transferred to 100 mL of synthetic ICI medium (Imperial Chemical Industries, Ltd., London, UK) (67) supplemented with 6 mM glutamine (Gln) and shaken under the same conditions for 2 days. Gene expression of the *FUM* cluster under the *tet^ON^* promoter was induced via the addition of 50 µg/mL doxycycline hyclate (Applichem, Darmstadt, Germany), and the cultures were shaken under the same conditions for an additional 5 days for FB_1_ and ceramide intermediate analyses.

### *F. verticillioides* growth assay on plates

*F. verticillioides* strains were first incubated on CM plates with an appropriate resistance marker at 30°C for 7 days from which spores were harvested and counted using a Cell Counter (Beckmann Coulter, Krefeld, Germany). 10 µL containing 10^4^ spores was spotted on the middle of fresh CM plates with and without supplementation of doxy (10–50 µg/mL). Each analyzed strain was spotted in triplicates. Plates were incubated for 3–6 days at 30°C. The colony diameters on plates containing doxy were adjusted by considering the growth of the strains in the absence of the inducer. The inhibition rate was then calculated with a formula: inhibition rate (IR) = (*C* − *T*)/*C* × 100 (68), where *C* (control) represents the average growth of the strain without doxy and *T* (treated) represents the growth of the respective strain and replicate with doxy. For gene expression analysis, strains were grown for 3 days on CM plates with or without 25 µg/mL doxy. Cells were covered with a layer of cellophane to enable harvesting and subsequent freeze-drying of the mycelium.

### Gene expression analysis via quantitative PCR

Fungal cultures were prepared as described above. Induction of 2-day-old ICI/6 mM Gln cultures with 10 µg/mL FB_1_ for 2 h was done previously, and expression analysis was essentially performed as previously described (8). Reactions were run on an Agilent Mx3000P qPCR System with the respective Agilent plastics (Santa Clara, CA, USA). Expression of the genes of interest and of the constitutively expressed reference genes (*FVEG_07930*/*FvACT* and *YFL039C*/*ScACT1* [69] encoding actin) was determined in triplicates with the primers listed in Table S1. Expression relative to actin was calculated using the ∆Ct method (70).

### Confocal microscopy

Microscopy experiments were performed on an Axio Observer Spinning Disc Confocal Microscope (Carl Zeiss, Jena, Germany) using 63×/1.2 oil or 100×/1.4 oil objectives with a numerical aperture (NA) value of 0.55. Laser lines of 488 nm and 561 nm were used for fluorophore excitation. Analysis was done with the ZEN 2.6 software, and in postprocessing, microscopy images were adjusted for brightness using the ImageJ software (https://imagej.nih.gov [71, 72]). Fungal hyphae were grown out from 10^4^ conidia in 300 µL of ICI/6 mM Gln as adherent cultures in 8-well dishes (ibidi, Gräfelfing, Germany) at 30°C for 16 h.

### Fum16 expression and purification

In order to render Fum16p soluble for bacterial expression, purification, and *in vitro* activity assay, its predicted N-terminal transmembrane domain had to be truncated; this corresponds to amino acids 1–88. Fum16p was expressed in *E. coli* BL21 (DE3) as a fusion construct N-terminally tagged with MBP under the control of an inducible T7 promoter (Fig. S6). A pre-culture was incubated overnight in LB medium containing 25 µg/mL kanamycin (37°C, 180 rpm). Then, 50 mL of Terrific Broth (TB) medium (73) was inoculated with the pre-culture to an OD_600_ of 0.1. The main culture was incubated (37°C, 180 rpm) until an OD_600_ of 0.5 was reached. Next, the temperature was lowered to 18°C, expression was induced after 1 h with 1 mM isopropyl β-d-1-thiogalactopyranoside (Carl Roth, Karlsruhe, Germany), and the culture was incubated overnight. The cell pellet was harvested by centrifugation (4,000 × *g*, 4°C, 20 min) and stored at −20°C until further processing.

Bacterial cells were thawed, resuspended in buffer A (20 mM Tris, 0.2 M NaCl, pH 8.0) containing 0.5 mM 4-(2-aminoethyl)benzenesulfonyl fluoride (AEBSF) and EDTA-free protease inhibitor cocktail (Roche, Grenzach-Wyhlen, Germany), and lysed using sonication (Sonopuls 2070, Bandelin, cycle 6, 75% intensity, 2 × 2 min) on ice. The protein preparations were centrifuged (16,000 × *g*, 4°C, 15 min), and the supernatants were applied to an MBP-Trap HP 1 mL column connected to an Aekta FPLC system (both GE Healthcare, Münich, Germany). After washing with 25 mM imidazole, the proteins were eluted with 500 mM imidazole using buffer B (20 mM Tris, 0.2 M NaCl, 10 mM maltose, pH 8.0). Protein-containing fractions were analyzed by Coomassie-stained SDS-PAGE, pooled, and concentrated via an Amicon filter (10 kDa cutoff) to 500 µL. This was applied to a Superdex 200 Increase 10/300 (Cytiva, Freiburg im Breisgau, Germany), and the protein was further purified by size-exclusion chromatography. Again, protein-containing fractions were analyzed (Fig. S6), pooled, flash-frozen in liquid nitrogen, and stored at −80°C. Protein concentrations were determined using the Bradford assay.

### Fum16 *in vitro* activity assay

The reaction was performed in a 100 µL volume containing 100 mM HEPES (pH 7.5), 0.5 mM TCEP, 2.5 mM MgCl_2_, 0.5 mM ATP, 0.5 mM CoA, 5 mM serine, 0.5 mM palmitic acid, 20 µg/mL FadD (purified in references 37, 74), 100 µg/mL SPT (purified in reference 74), and 100 µg/mL Fum16p. The reaction was incubated overnight at 37°C and extracted with 50% (vol/vol) methanol and filtered through a 0.2 µm PTFE filter (Chromafil, Macherey-Nagel, Düren, Germany). The filtrate was then subjected to HPLC-HRMS analysis. The negative control did not contain any SPT enzyme.

### *S. cerevisiae* cell viability assay

FB_1_ growth inhibition of *S. cerevisiae* strains expressing *F. verticillioides* genes *FUM8*, *FUM15*, *FUM16*, and *FUM18* was evaluated by monitoring the OD_600_ every 15 min for up to 24 h. The experiments were conducted in an Infinite M200 plate reader (Tecan, Crailsheim, Germany) with either sterile black 96-well plates (BRAND plates; VWR, Darmstadt, Germany), or sterile black 24-well plates (ibidi, Gräfelfing, Germany). Incubation was performed with SD-Ura at 30°C. Each well contained either 500 µg/mL FB_1_ (Cayman Chemicals, Ann Arbor, MI) or water as a control, and 0.1 OD_600_ cells diluted in SD-Ura.

### FB_1_ measurement for *F. verticillioides* liquid cultures

The supernatant was separated from mycelium through Miracloth and further clarified via centrifugation. Then, 100 µL of the clarified supernatant was mixed with 100% (vol/vol) MeOH and 1% (vol/vol) naringenin as an internal standard (1 mg/mL in methanol; Sigma-Aldrich, Steinheim, Germany) to give 1 mL (thereby diluted 1:10) and filtered through a 0.2 µm PTFE filter.

Filtered mycelia were washed with water, lyophilized, and weighed. Approximately 100 mg of dry mycelium was powdered using liquid nitrogen and resuspended in 1 mL MeOH:CHCl_3_ (1:1, vol/vol). The mycelium suspension was vigorously shaken (1,400 rpm) at 40°C overnight. Cell debris were pelleted (16,000 × *g*, 4°C, 15 min), and the supernatants were transferred to a fresh tube. The pellets were resuspended again in 1 mL MeOH:CHCl_3_ (1:1, vol/vol) and shaken for 1 h. The process was repeated two more times. The combined supernatants were collected, evaporated using SpeedVac, and resuspended overnight in 100% (vol/vol) MeOH. The samples were then filtered with a 0.2 µm PTFE filter prior to HPLC-HRMS analysis.

### FB_1_ measurement for *S. cerevisiae* liquid cultures

The whole content from each well of the cell viability assay plate (see above) was centrifuged (10,000 × *g*, 4°C, 5 min) and 100 µL supernatant was mixed with 100% (vol/vol) MeOH to give 1 mL and filtered through a 0.2 µm PTFE filter (thereby diluted 1:10).

### Extraction of ceramide intermediates from *F. verticillioides* and *S. cerevisiae*

For *F. verticillioides*, the same extraction method was used as described above for FB_1_ extraction from mycelium. For *S. cerevisiae* cells, the obtained pellet (10,000 × *g*, 4°C, 5 min) was washed twice with 900 µL water and resuspended in 1 mL MeOH:CHCl_3_ (1:1, vol/vol). The suspension was mixed with 0.5 mm glass beads, and the cells were lysed using a SpeedMill Plus (Analytic Jena, Jena, Germany, two cycles of 1 min). Cell debris were pelleted (10,000 × *g*, 4°C, 10 min), and the supernatant was collected. The pellet was resuspended again in 1 mL MeOH:CHCl_3_ (1:1, vol/vol) and lysed once more. The process was repeated once more. The combined supernatants were collected, evaporated using SpeedVac, and resuspended overnight in 100% (vol/vol) MeOH. The samples were then filtered with a 0.2 µm PTFE filter for HPLC-HRMS analysis.

### HPLC-HRMS analysis

HPLC-HRMS analysis was performed using an LC-MS system consisting of a Q-Exactive Plus Hybrid Quadrupole Orbitrap mass spectrometer using electrospray ionization and a Dionex UltiMate 3000 UHPLC system (Thermo Fisher Scientific, Dreieich, Germany). Sample separation via HPLC was performed with a Kinetex C_18_ column (2.1 × 150 mm, 2.5 µm, 100 Å, Phenomenex) at a flowrate of 0.3 mL/min and column oven at 40°C. For analysis of FB_1_ and ceramide intermediates from both *F. verticillioides* and *S. cerevisiae*, an injection volume of 3 µL was set. The following elution gradient was used for FB_1_ detection (solvent A, H_2_O plus 0.1% [vol/vol] HCOOH; solvent B, acetonitrile plus 0.1% [vol/vol] HCOOH): 5% B for 0.5 min, 5 to 97% B in 11.5 min, and 97% B for 3 min. The following gradient was used for ceramide intermediates detection: 5% B for 0.5 min, 5 to 97% B in 54.5 min, and 97% B for 3 min. Raw LC-MS data were analyzed using XCalibur (Thermo Fisher Scientific, Dreieich, Germany) with a mass resolution of 10 ppm (Fig. S3 and S10). FB_1_ peak areas were normalized against the internal standard to account for variability between runs. FB_1_ and ceramide intermediate levels were related to the dry weight of the *F. verticillioides* cultures, which were performed in biological triplicate. The standard curve was prepared by injecting 3 µL of the following FB_1_ concentrations prepared in ethanol in triplicate: 0.1 µg/mL, 0.5 µg/mL, 1 µg/mL, 10 µg/mL, and 100 µg/mL.

## SUPPLEMENTAL MATERIAL

Supplemental material mbio.02681-24-s0001.pdf Supplemental figures and table.

ASM does not own the copyrights to Supplemental Material that may be linked to, or accessed through, an article. The authors have granted ASM a non-exclusive, world-wide license to publish the Supplemental Material files. Please contact the corresponding author directly for reuse.
