# Supplementary material for: The palmitoyl-CoA ligase Fum16 is part of a Fusarium verticillioides fumonisin subcluster involved in self-protection
Source: mBio. 2024 Dec 20;16(2):e02681-24. doi: 10.1128/mbio.02681-24 (PMC11796371; doi:10.1128/mbio.02681-24)
Supplement: Supplemental material — Supplemental figures and table. [file mbio.02681-24-s0001.pdf]

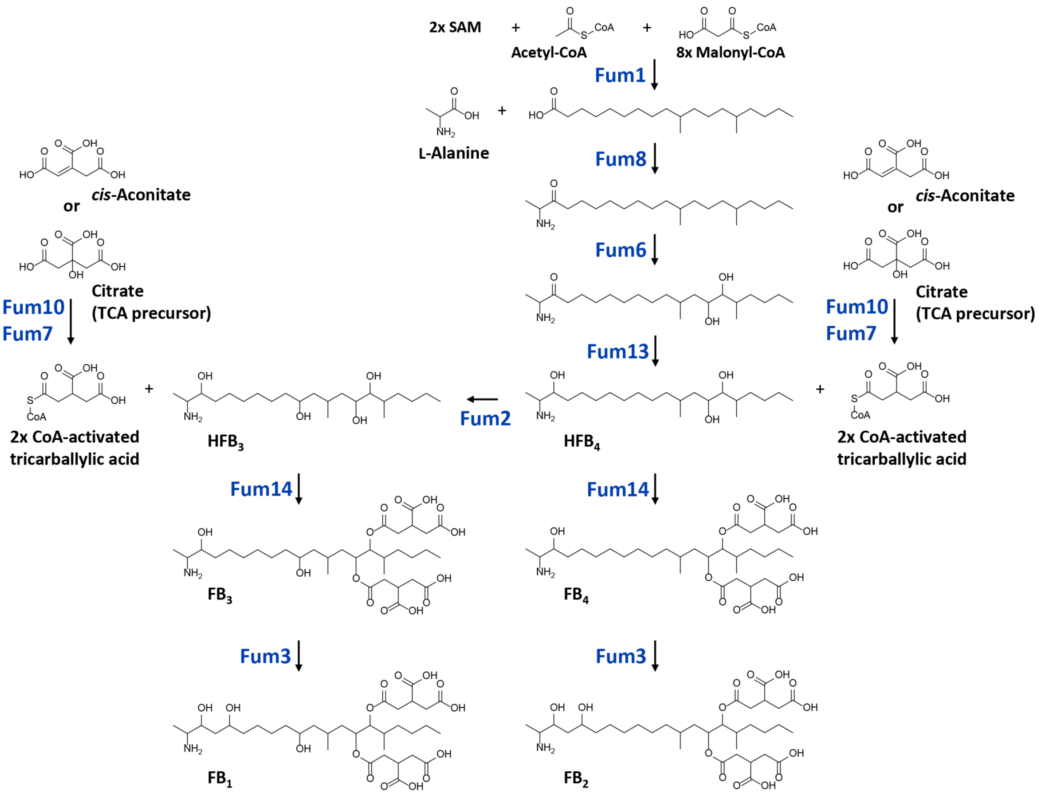

Supplementary Figure 1. Fumonisin biosynthetic pathway. The PKS Fum1 and aminotransferase Fum8 condense the first biosynthetic intermediate, which is thereafter modified by cluster-encoded enzymes. Fum10 and Fum7 provide a CoA-activated tricaballylic acid moiety which is based on a precursor from the tricarboxylic acid (TCA) cycle. Hydrolyzed FB (HFB) refers to the backbone without the tricarballylic acid side chains. FB_1 is the major biosynthetic product in *F. verticillioides*.

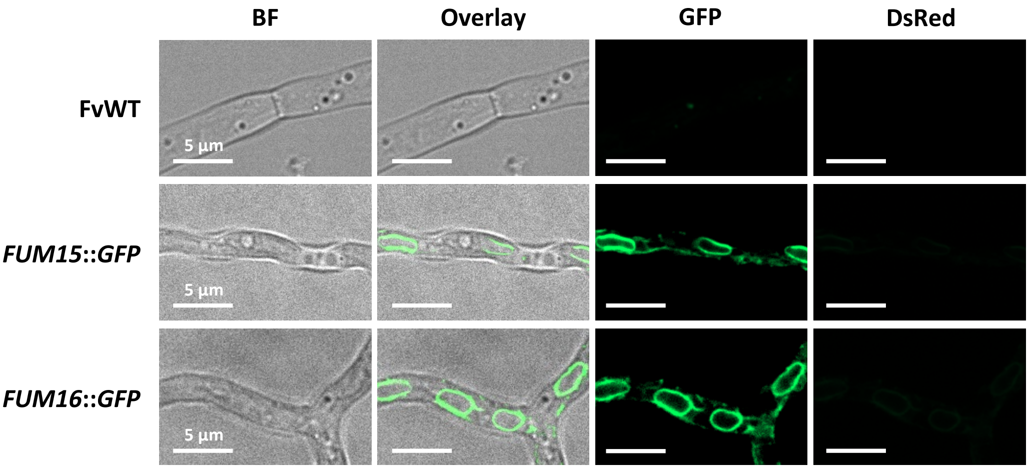

Supplementary Figure 2. Confocal microscopy for localization of Fum15-GFP and Fum16-GFP, using *F. verticillioides* WT as control. Conidia were inoculated in ICI/6 mM Gln and grown as a standing culture overnight. Indicated strains were analyzed for GFP and DsRed fluorescence. Shown are individual channels in black/white, brightfield (BF) and an overlay with BF in color.

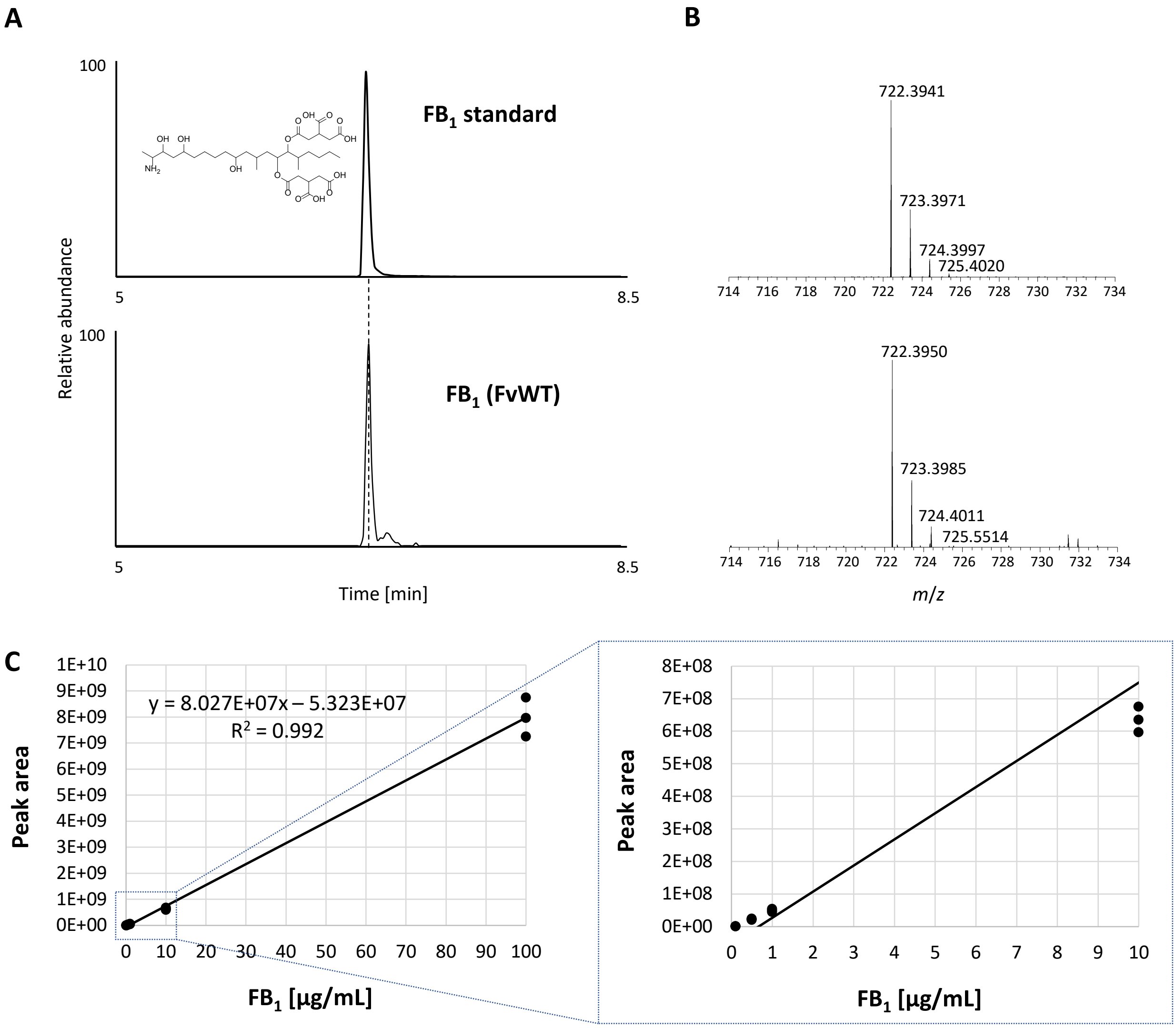

Supplementary Figure 3. HPLC-HRMS analysis of FB_1, comparing the standard with the supernatant of the *F. verticillioides* WT. A) The extracted ion chromatograms for *m/z* = 722.3957 ± 10 ppm, corresponding to $[M+H]^+$ of FB_1. **B)** Measured accurate masses of the isotopic distribution. **C)** Standard curve with 0.1 – 100 µg/mL FB_1. Injection volume was 3 µL, same as for unknown FB_1-containing samples.

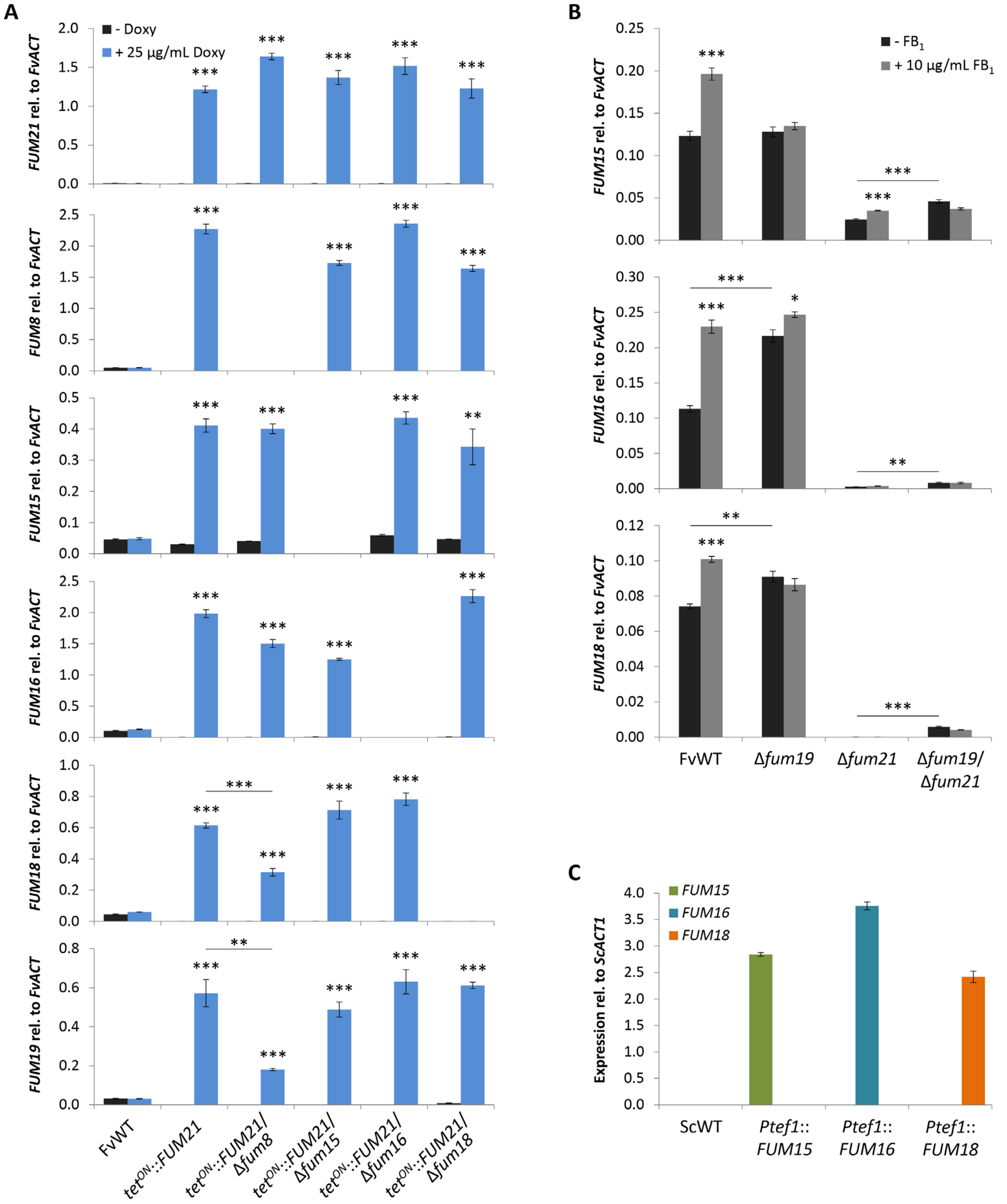

Supplementary Figure 4. Expression of *FUM* cluster genes in *tet*^{ON}::*FUM21* mutants, in response to added FB$_1$, and in the heterologous host *S. cerevisiae*. A) *F. verticillioides* strains were grown for 3 days on solid complete medium with and without 25 µg/mL doxycycline (doxy). **B)** Strains were grown for 2 days in liquid ICI/6 mM Gln prior to induction for 2 hours with 10 µg/mL FB$_1$ (Janevska *et al.*, 2020). **C)** *S. cerevisiae* plasmid-harboring strains (control with pYES2::*Ptef1*) were grown in liquid SD-Ura to OD$_{600}$ of 1. Transcript levels (means ± SD, n = 3) were compared between WT and mutants (A), between treatments (B) or as indicated, using *t*-test; $P < 0.05$ (*), $P < 0.01$ (**), $P < 0.001$ (***).

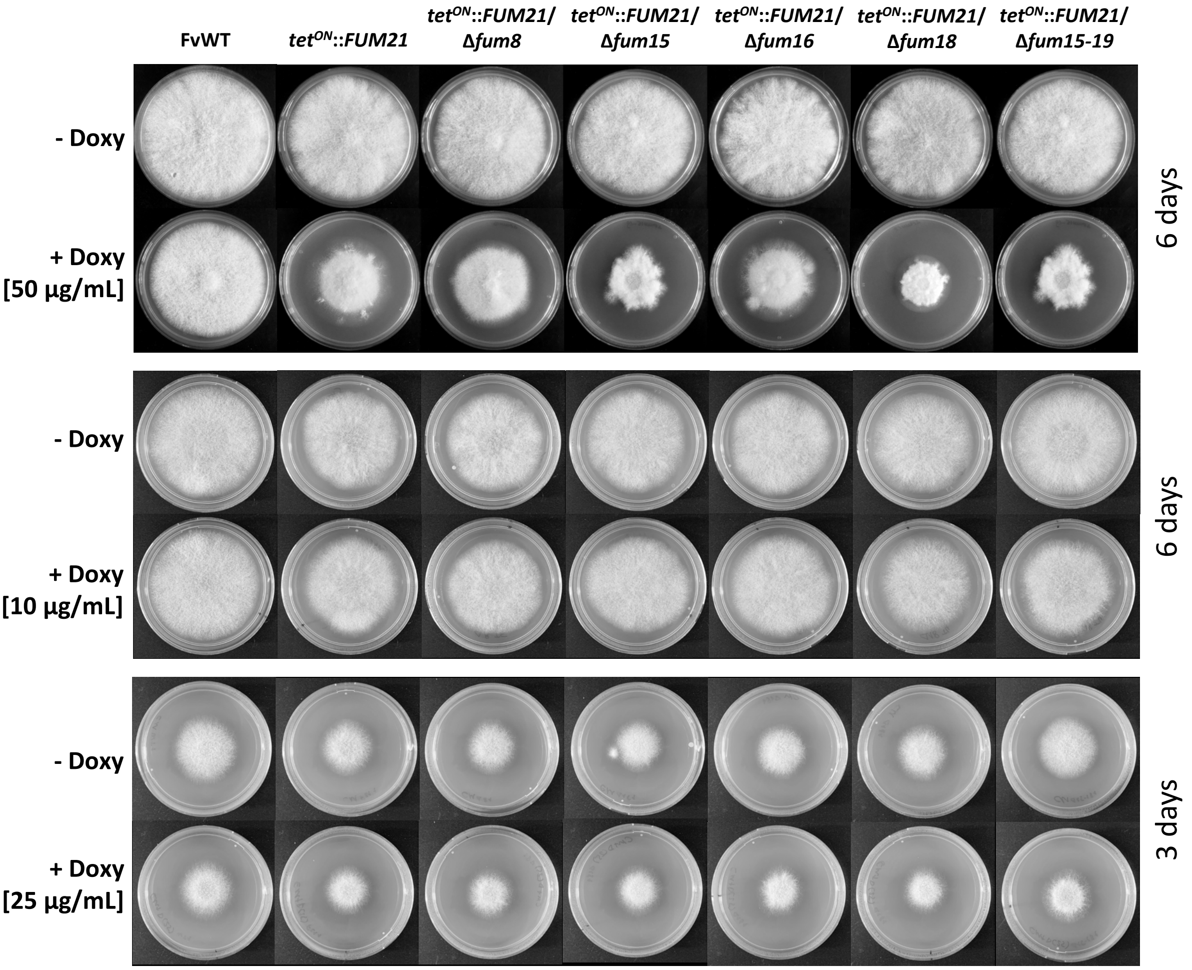

Supplementary Figure 5. Analysis of the phenotype of *tet*^{ON}::*FUM21* and deletion mutants thereof using different incubation times as well as concentrations of the inducer. Growth assay of 3 and 6 days performed on complete medium plates under uninduced and induced (10, 25, and 50 µg/mL doxy) conditions (*n* = 3).

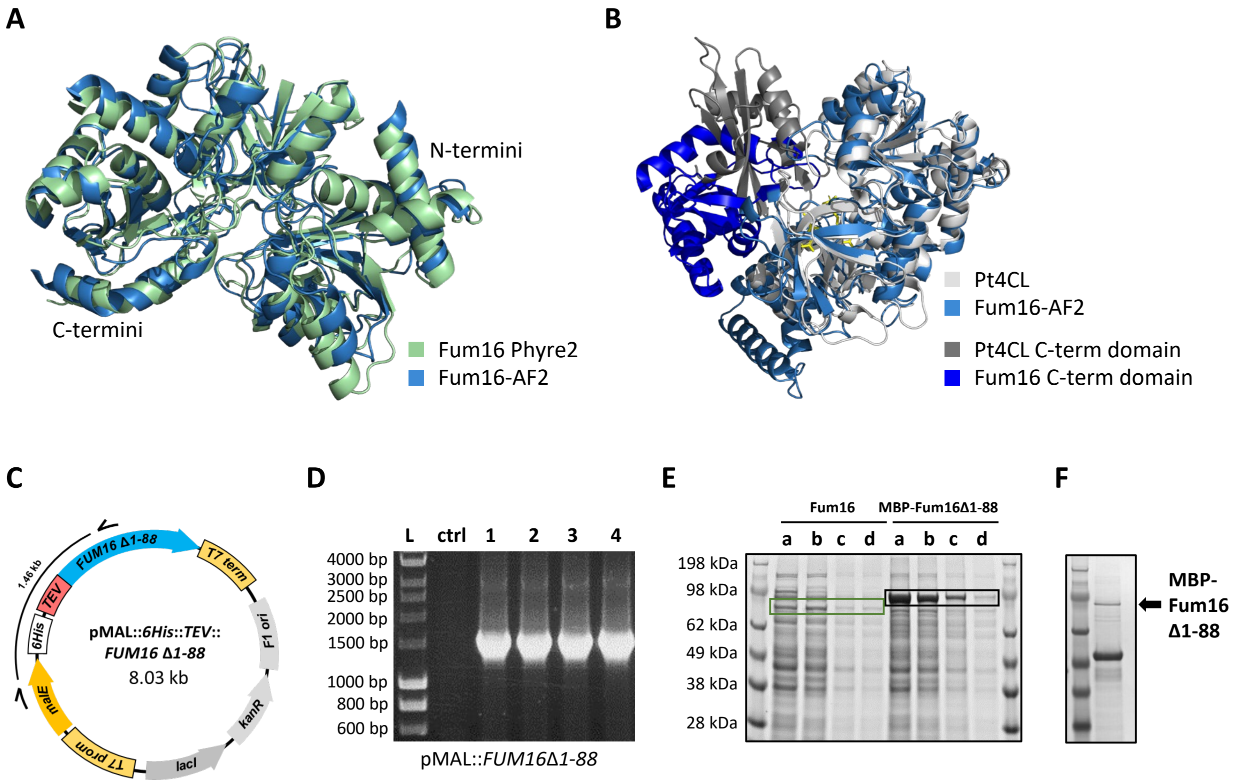

Supplementary Figure 6. Fum16 modeling, expression and purification. A) Alignment of Fum16 homology prediction (Phyre2, light green) with Fum16-AF2 (cyan). The RSMD is 1.840. The first 88 amino acids are omitted in the alignment. **B**) Alignment of Fum16-AF2 (cyan) with the *Populus tormentosa* 4-coumarate-CoA ligase (Pt4CL, gray, PDB entry 3ni2) in complex with adenosine phenyl-propyl-phosphate (APP, yellow). The RSMD is 1.505. The structures display higher variability in their C-terminal domains (dark blue and dark gray). **C**) Plasmid used to express MBP-Fum16 in *E. coli* BL21 (DE3). **D**) Diagnostic PCR of *E. coli* BL21 (DE3) harboring pMAL::*6His*::*TEV*::*FUM16Δ1-88*. **E**) Coomassie-stained SDS-PAGE gel of the expression tests using full-length, and MBP-tagged and truncated Fum16. Shown are a) the lysate and b) the soluble fraction after induced expression with IPTG, and c) the lysate and d) soluble fraction after expression in auto-induction medium. The band corresponding to full-length Fum16 is indicated with a green box (theoretical MW: 75 kDa), while MBP-Fum16Δ1-88 is indicated with a black box (theoretical MW: 108 kDa). **F**) Coomassie-stained SDS-PAGE gel of the concentrated elution fraction. MBP-Fum16Δ1-88 is indicated with a black arrow (theoretical MW: 108 kDa).

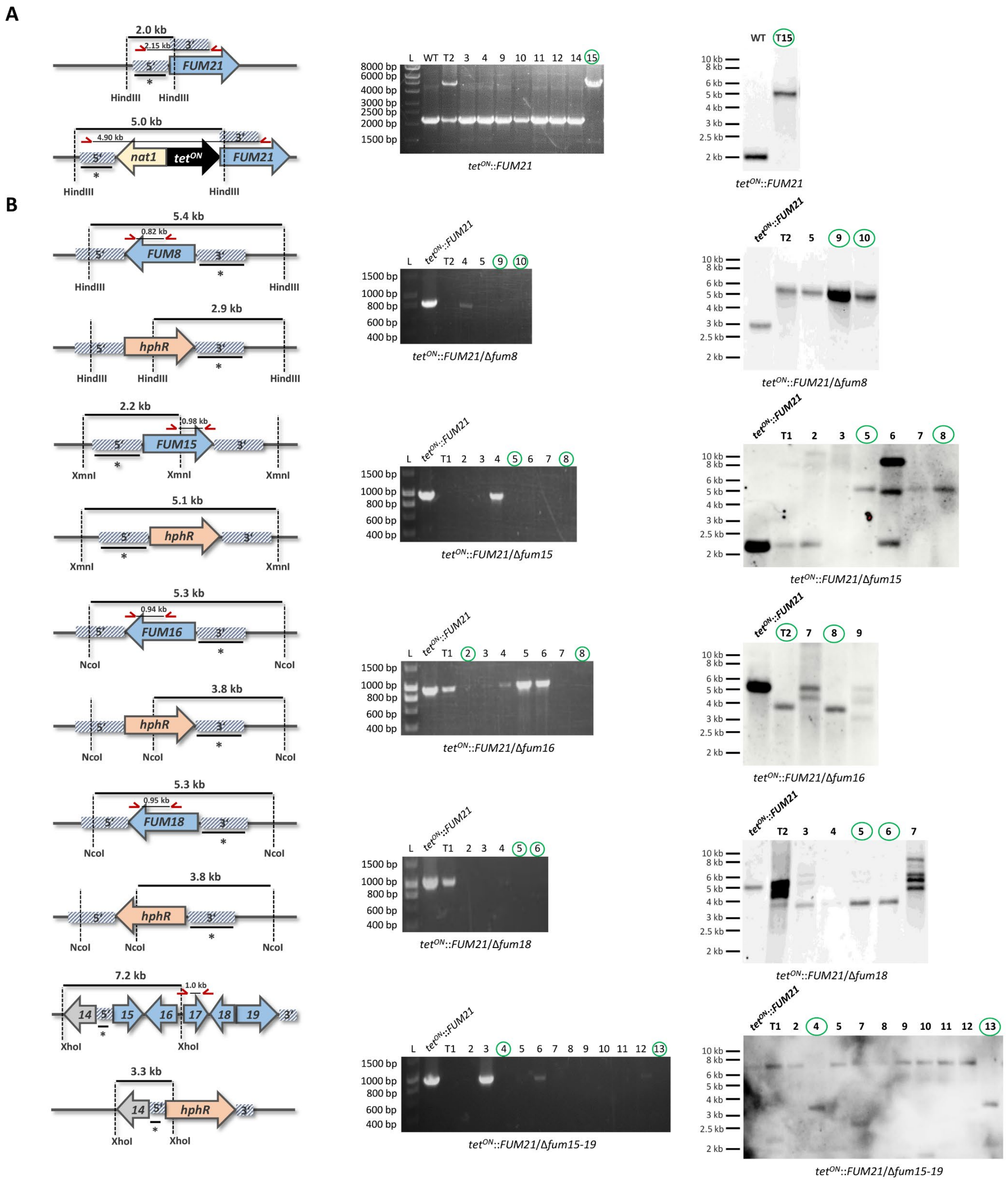

Supplementary Figure 7. Verification of *F. verticillioides tetON*::*FUM21* mutant strains. Schematics, diagnostic PCRs and Southern blots for the generation of the *F. verticillioides* mutants used in this study. A) *tetON*::*FUM21* strategy via homologous recombination with the nourseothricin resistance cassette (*nat1*). **B)** Single gene deletions in the *tetON*::*FUM21* strain via homologous recombination with the hygromycin B resistance cassette (*hphR*) for *FUM8*, *FUM15*, *FUM16*, *FUM18*, and *FUM15-19*. Primers for diagnostic PCRs are indicated with half-arrow pairs (see Supplementary Table 1). For Southern blots, probes were PCR amplified using primers indicated in Supplementary Table 1 and are marked with (*). Positive transformants used in this study are highlighted with a green circle.

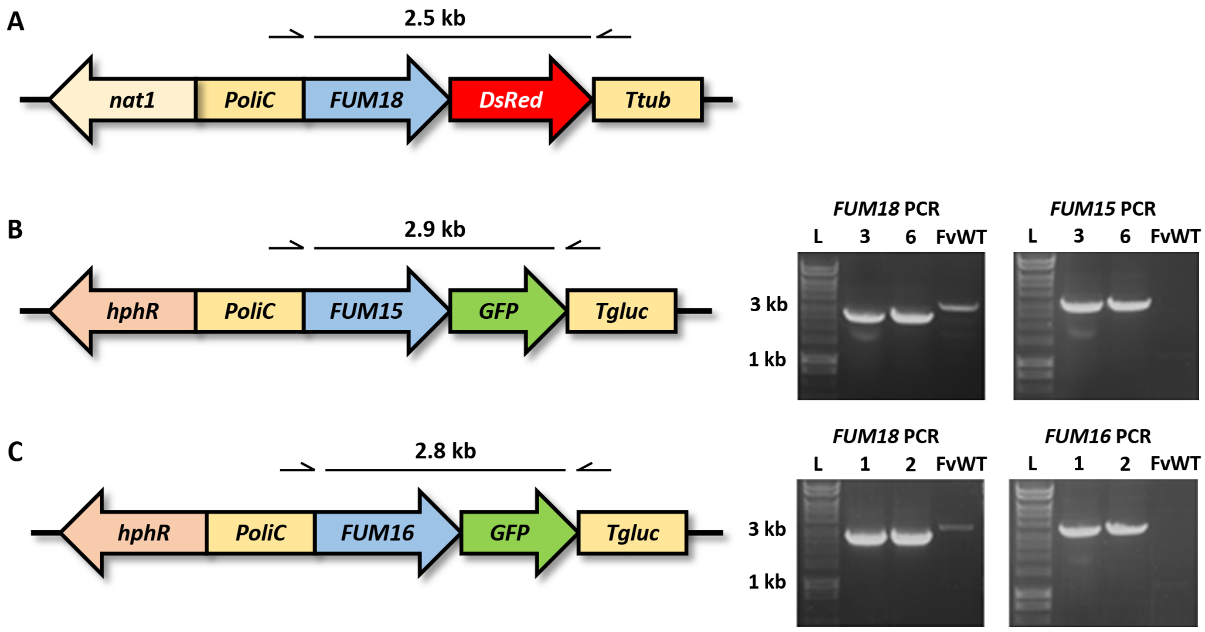

Supplementary Figure 8. PCR verification of *F. verticillioides* microscopy strains. Verification via diagnostic PCR of **A)** the acceptor strain *FUM18*::*DsRed* (Janevska *et al*., 2020), and double mutants **B)** *FUM18*::*DsRed*/*FUM15*::*GFP*, and **C)** *FUM18*::*DsRed*/*FUM16*::*GFP*. Primer pairs are indicated by black half arrows. *F. verticillioides* M-3125 was used as WT control.

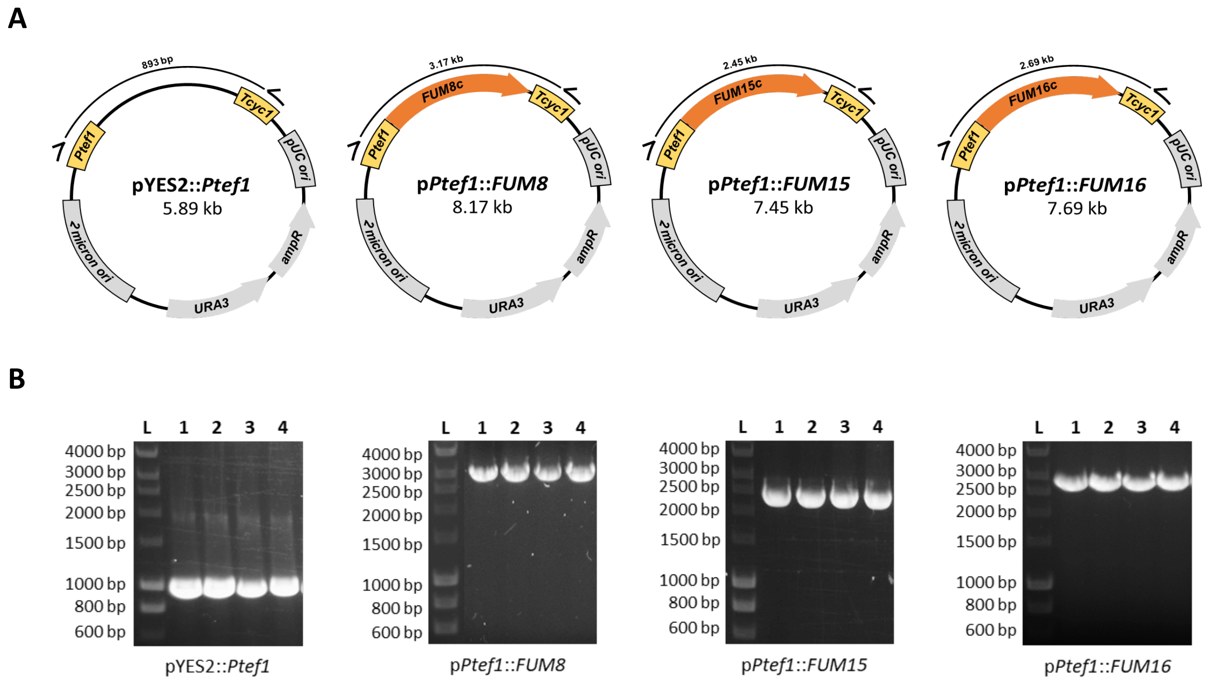

Supplementary Figure 9. PCR verification of *S. cerevisiae* expression strains. A) *F. verticillioides* intron-less genes from the fumonisin cluster were expressed under control of the constitutive yeast promoter *Ptef1*. pYES2::*Ptef1* was used for cloning and served as empty vector control. Primer pairs for diagnostic PCR are indicated by black half arrows. **B)** Diagnostic PCRs of *S. cerevisiae* BY4741 harboring pYES2::*Ptef1*, p*Ptef1*::*FUM8*, p*Ptef1*::*FUM15*, and p*Ptef1*::*FUM16*.

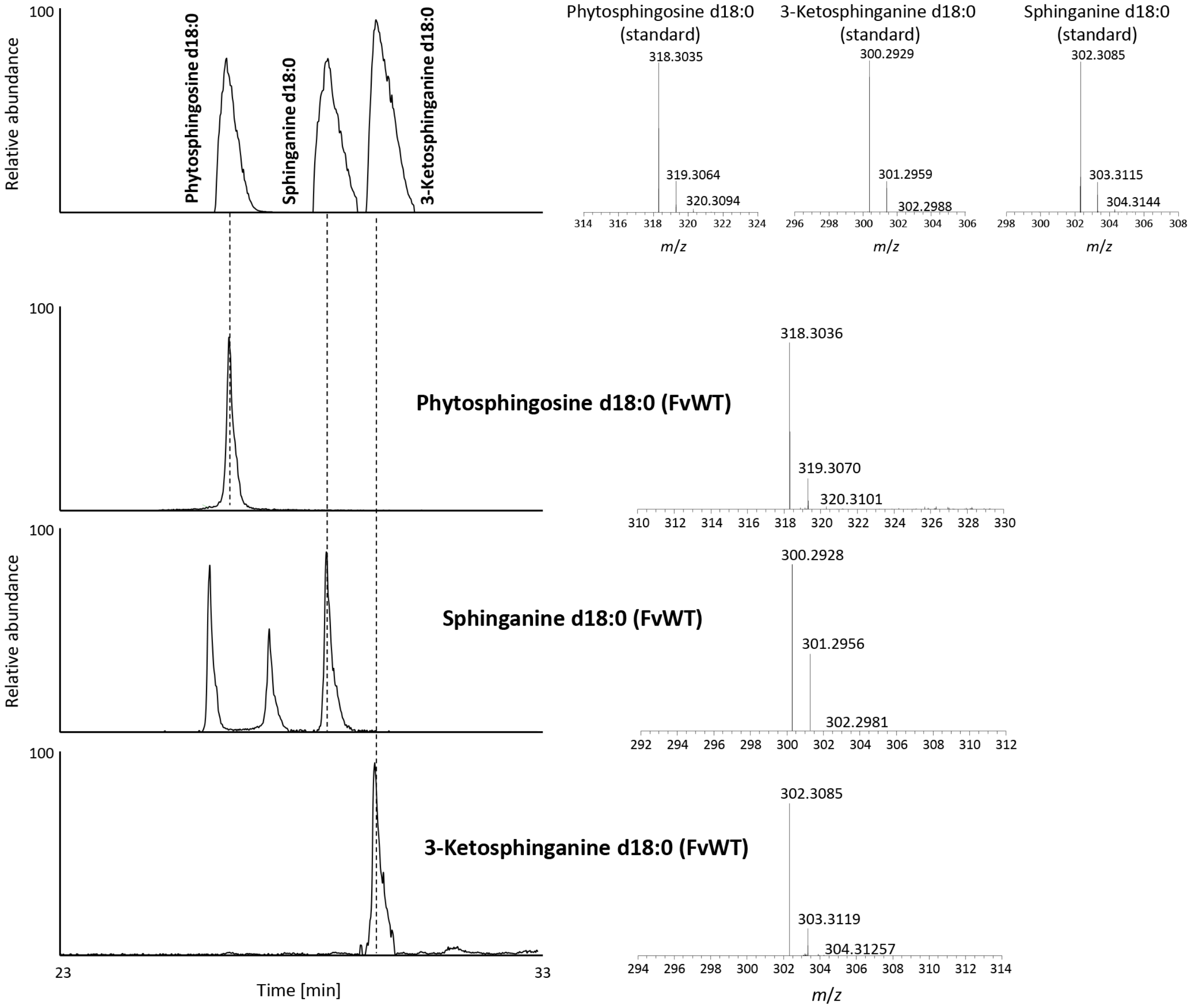

Supplementary Figure 10. HPLC-HRMS analysis of ceramide intermediates, comparing the standards with the mycelium extract of the *F. verticillioides* WT. Presented are the extracted ion chromatograms for *m/z* = 318.3003 ± 10 ppm, *m/z* = 300.2897 ± 10 ppm, and *m/z* = 302.3054 ± 10 ppm, corresponding to $[M+H]^+$ of phytosphingosine, sphinganine, and 3-ketosphingosine, respectively (left), and the measured accurate masses of the isotopic distribution (right).

Supplementary Table 1. Primers used in this study.

Gene / Construct	Primer	Sequence 5´ → 3´
***F. verticillioides* overexpression and deletion mutants**		
tet^{ON}***::FUM21***	fum21_5F	GTAATACGACTCACTATAGGGAATATTGTTCATATCGGGACATCCAG
	nat1_fum21_5R	CTCTACATGAGCATGCCCTGCCCCTGACGCAAAAGTTGATGATGAAATAA
	TETon_FUM21_3F	CCTCCATCTCAACTCCATCACATCACAATGGCGGGGTCCATTGTGTTTG
	TetON_FUM21_3R2	GACATAACTAATTACATGATGCGGCCCCTCGAACACTGTTATTGTGTTAG
	nat1_tetON_F2	AGTGCTCCTTCAATATCATCTTCTGTCCTTTGCCCGGTGTATGAAACCGG
	gpda_for_vv	CTTGGTTGAATTTAGAACGTGG
tet^{ON}***::FUM21/ Δfum8***	d_fum8_5F	GTAATACGACTCACTATAGGGAATATTATCCATCCCCGTTATAAATGTGC
	d_fum8_3R	GACATAACTAATTACATGATGCGGCCCTCATTCACGGCACTGGCGGGAAC
tet^{ON}***::FUM21/ Δfum15***	d_fum15_5F	GTAATACGACTCACTATAGGGAATATTATTGGTCCGGAACGGTTTTCGCC
	d_fum15_hyg_5R	TAGTGCCACGTTCTAAATTCAACCAAGACTGCCTGTTCTAGGATTGTCAA
	d_fum15_hyg_3F	AGCACTCGTCCGAGGGCAAAGGAATAGCGCTGGTAATGGCAAGTTTGATG
	d_fum15_3R	GACATAACTAATTACATGATGCGGCCCACTGCGGTATATCCTCAACGGC
tet^{ON}***::FUM21/ Δfum16***	d_fum16_5F	GTAATACGACTCACTATAGGGAATATTCTTGTAAAAGGTAATAGAATTTG
	d_fum16_5R	TAGTGCCACGTTCTAAATTCAACCAAGGCTGTGAGAGTTTCTCTTCCC
	d_fum16_3F	AGCACTCGTCCGAGGGCAAAGGAATAGATTAACGGGTTCGGTTGAGAGGG
	d_fum16_3R	GACATAACTAATTACATGATGCGGCCCATCAAACGCCAAAAAGCTGGAG
tet^{ON}***::FUM21/ Δfum18***	fum18_5F	GTAATACGACTCACTATAGGGAATATTCAGACTGTACACTCCACTGG
	fum18_5R	TAGTGCCACGTTCTAAATTCAACCAAGAAAGATTAATAAACGTCAAG
	fum18_3F	AGCACTCGTCCGAGGGCAAAGGAATAGGATAGTGAGATATTAGAAAAC
	fum18_3R	GACATAACTAATTACATGATGCGGCCCGAAGAGGCTTCTGCGGCCTC
tet^{ON}***::FUM21/ Δfum15-19***	d_fum15_5F	GTAATACGACTCACTATAGGGAATATTATTGGTCCGGAACGGTTTTCGCC
	d_fum15_hyg_5R	TAGTGCCACGTTCTAAATTCAACCAAGACTGCCTGTTCTAGGATTGTCAA
	fum19_3F	AGCACTCGTCCGAGGGCAAAGGAATAGATAATATGTAGGATCGATCC
	fum19_3R	GACATAACTAATTACATGATGCGGCCCCTAGAAATGCCCTTCGGCAC
hphR	gpda_for_vv	CTTGGTTGAATTTAGAACGTGG
	Hph_Rev_VV2	CTATTCCTTTGCCCTCGGACGAG
natR	hph_trpC_F	GACAGAAGATGATATTGAAGGAGC
	nat1_R	TCAGGGGCAGGGCATGCTCA
**Southern Blot probes for deletions**		
tet^{ON}***::FUM21***	fum21_5F	GTAATACGACTCACTATAGGGAATATTGTTCATATCGGGACATCCAG
	nat1_fum21_5R	CTCTACATGAGCATGCCCTGCCCCTGACGCAAAAGTTGATGATGAAATAA
tet^{ON}***::FUM21/ Δfum8***	d_fum8_5F	GTAATACGACTCACTATAGGGAATATTATCCATCCCCGTTATAAATGTGC
	d_fum8_at2_5R2	AATGCTCCTTCAATATCATCTTCTGTCATGTAACTAGGCAGAGTAGTAG
tet^{ON}***::FUM21/ Δfum15***	d_fum15_5F	GTAATACGACTCACTATAGGGAATATTATTGGTCCGGAACGGTTTTCGCC
	d_fum15_hyg_5R	TAGTGCCACGTTCTAAATTCAACCAAGACTGCCTGTTCTAGGATTGTCAA
tet^{ON}***::FUM21/ Δfum16***	d_fum16_5F	GTAATACGACTCACTATAGGGAATATTCTTGTAAAAGGTAATAGAATTTG
	d_fum16_5R	TAGTGCCACGTTCTAAATTCAACCAAGGCTGTGAGAGTTTCTCTTCCC
tet^{ON}***::FUM21/ Δfum18***	fum18_5F	GTAATACGACTCACTATAGGGAATATTCAGACTGTACACTCCACTGG
	fum18_5R	TAGTGCCACGTTCTAAATTCAACCAAGAAAGATTAATAAACGTCAAG
tet^{ON}***::FUM21/ Δfum15-19***	fum15/16_5F	GTAATACGACTCACTATAGGGAATATTATTGGTCCGGAACGGTTTTCGC
	fum15/16_5R	TAGTGCCACGTTCTAAATTCAACCAAGACTGCCTGTTCTAGGATTGTC
**Diagnostic PCRs for deletions**		
tet^{ON}***::FUM21***	fum21_5F	GTAATACGACTCACTATAGGGAATATTGTTCATATCGGGACATCCAG
	TetON_FUM21_3R2	GACATAACTAATTACATGATGCGGCCCCTCGAACACTGTTATTGTGTTAG
tet^{ON}***::FUM21/ Δfum8***	d_fum8_at1_diag	GAGTATGTCACACCCAGCTTGG
	fum8_Seq_F	GAGGAACTGGCCACATACTTGGG
tet^{ON}***::FUM21/ Δfum15***	d_fum15_5diag	GAGAGTTTGCGACAGTTCTC
	d_fum15_3diag	GCCTCAGTTCGACCACGGAC
tet^{ON}***::FUM21/ Δfum16***	d_fum16_5diag	TATCCCTTGAAGACTGCTGG
	d_fum16_3diag	CGAGCACCCGCAATTCAACG
tet^{ON}***::FUM21/ Δfum18***	fum18_F	GGCTTCACGTTACTGGCTGGTG
	fum18_R	GATCCATGGCTCAATCAAGTGCC
tet^{ON}***::FUM21/ Δfum15-19***	fum17_F	CAGACCCGGCCAAGGAAACAG
	fum17_R	GCTCGAAAGGACCAACGGTACG

F. verticillioides **microscopy mutants**		
FUM15*::*GFP	pNDH_fum15_GFP_fw	AACTCCATCACATCACAATCGATCCAAATGCGTGGTCTGAACAATATTG
	pNDH_fum15_GFP_rv	ATTACTTACCTCACCCTTGGAAACCATTACAACGGCTATTGGCCTCAC
FUM16*::*GFP	pNDH_fum16_GFP_fw	AACTCCATCACATCACAATCGATCCAAATGTATCATACAGTGCCCTATAC
	pNDH_fum16_GFP_rv	ATTACTTACCTCACCCTTGGAAACCATTCCAATGCTTTGCATACAC
GFP	pNDH_GFP_F	ATGGTTTCCAAGGGTGAGGT
	eGFP_STOP-Tgluc_Rv	CCTAATCATACATCTTATCTACATACGCTAAGCGGCCGCTTTGTAAAGTT
**Diagnostic PCR**	PoliC_Seq_F2	GGGAGACGTATTTAGGTGCTAGGG
	Tgluc_Seq_R2	CCGCCCTCTTTTGTCTTCCGC
S. cerevisiae **expression strains**		
FUM8	fum8_Tef2_F	CAATCTAATCTAAGTTTTAATTACAAAATGTCCACCCAGGAAATCACTAC
	Fum8-cyc1t_Rv	AGCGTGACATAACTAATTACATGATTCAACATGTCCCTCGCGATA
FUM15	fum15_Tef2_F	CAATCTAATCTAAGTTTTAATTACAAAATGCGTGGTCTGAACAATATTGC
	fum15_Tef2_R	GTGACATAACTAATTACATGATGTTTAAACCTATACAACGGCTATTGGCC
FUM16	fum16_Tef2_F	CAATCTAATCTAAGTTTTAATTACAAAATGTATCATACAGTGCCCTATAC
	Fum16-cyc1t_Rv	AGCGTGACATAACTAATTACATGATTCATCCAATGCTTTGCATACACT
**Linearized vector**	TEF_Rv	TTTGTAATTAAAACTTAGATTAGATTGCTATGC
	pYes2_cyc1T_Fw	ATCATGTAATTAGTTATGTCACGCTTACATTCACG
**Diagnostic PCR**	Tef1p_fw	CAAAATGTTTCTACTCCTTTTTTACTCTTCCAG
	CYC1-R	GCAAATTAAAGCCTTCGAGCGTC
E. coli **overexpression strain**		
FUM16	pMAL_Fum16short_fw	TCTGGTGAAAACCTGTATTTCCAAGGATCCGGCCCTTACAATTACTTGAG
	pMAL_Fum16short_rv	GTTGTAAAACGACGGCCAGTGCCAAGCTTTCATCCAATGCTTTGCATAC
**Diagnostic PCR**	MBP_seq_Fw	TTGCCGCCACTATGGAAAACGCCCA
	d_fum16_5diag	TATCCCTTGAAGACTGCTGG
**RT-qPCR**		
FUM8	fum8_qRT_F	GCGCTTGAGAGACGACTGGCC
	fum8_qRT_R	GGTTGGCGCATGCACTGAGC
FUM15	fum15_qRT_F	CGTTGGTTATCACCCAAGAACGGG
	fum15_qRT_R	GCCTCAGTTCGACCACGGAC
FUM16	fum16_qRT_F	CACAGATGCGGACGCTGGC
	fum16_qRT_R	CAAACTCGCCAATGTCGCCTG
FUM18	fum18_qRT_F	CGCCATTGGTGTCTTGGATGG
	fum18_qRT_R	GAGATTGCTGCCAGCCAGAACC
FUM19	fum19_qRT_F	CACAGCGTAGGGCAGGTTAGCG
	fum19_qRT_R	TCCAGAAGCTGAGCTGGATGGC
FUM21	fum21_qRT_F	CGGCGTCAACGTGTGCAGAG
	fum21_qRT_R	CTCAATCGGACACCTGCACCG
FoACT	FoACT_qRT_F	ATGTCACCACCTTCAACTCCA
	FoACT_qRT_R	CTCTCGTCGTACTCCTGCTT
ScACT1	ScACT1_qRT_F	TTCCCAGGTATTGCCGAAA
	ScACT1_qRT_R	TTGTGGTGAACGATAGATGGA
